# Gut microbiota variation of a tropical oil-collecting bee species far exceeds that of the honeybee

**DOI:** 10.3389/fmicb.2023.1122489

**Published:** 2023-05-17

**Authors:** Elif Kardas, Angie M. González-Rosario, Tugrul Giray, James D. Ackerman, Filipa Godoy-Vitorino

**Affiliations:** ^1^Department of Biology, University of Puerto Rico, San Juan, PR, United States; ^2^Department of Microbiology and Medical Zoology, School of Medicine, University of Puerto Rico, San Juan, PR, United States

**Keywords:** gut microbiota, sociality, mother bee, pollen provision, oil-collecting bee

## Abstract

**Introduction:**

Interest for bee microbiota has recently been rising, alleviating the gap in knowledge in regard to drivers of solitary bee gut microbiota. However, no study has addressed the microbial acquisition routes of tropical solitary bees. For both social and solitary bees, the gut microbiota has several essential roles such as food processing and immune responses. While social bees such as honeybees maintain a constant gut microbiota by direct transmission from individuals of the same hive, solitary bees do not have direct contact between generations. They thus acquire their gut microbiota from the environment and/or the provision of their brood cell. To establish the role of life history in structuring the gut microbiota of solitary bees, we characterized the gut microbiota of *Centris decolorata* from a beach population in Mayagüez, Puerto Rico. Females provide the initial brood cell provision for the larvae, while males patrol the nest without any contact with it. We hypothesized that this behavior influences their gut microbiota, and that the origin of larval microbiota is from brood cell provisions.

**Methods:**

We collected samples from adult females and males of *C. decolorata* (*n* = 10 each, *n* = 20), larvae (*n* = 4), and brood cell provisions (*n* = 10). For comparison purposes, we also sampled co-occurring female foragers of social *Apis mellifera* (*n* = 6). The samples were dissected, their DNA extracted, and gut microbiota sequenced using 16S rRNA genes. Pollen loads of *A. mellifera* and *C. decolorata* were analyzed and interactions between bee species and their plant resources were visualized using a pollination network.

**Results:**

While we found the gut of *A. mellifera* contained the same phylotypes previously reported in the literature, we noted that the variability in the gut microbiota of solitary *C. decolorata* was significantly higher than that of social *A. mellifera*. Furthermore, the microbiota of adult *C. decolorata* mostly consisted of acetic acid bacteria whereas that of *A. mellifera* mostly had lactic acid bacteria. Among *C. decolorata*, we found significant differences in alpha and beta diversity between adults and their brood cell provisions (Shannon and Chao1 *p* < 0.05), due to the higher abundance of families such as Rhizobiaceae and Chitinophagaceae in the brood cells, and of Acetobacteraceae in adults. In addition, the pollination network analysis indicated that *A. mellifera* had a stronger interaction with *Byrsonima* sp. and a weaker interaction with *Combretaceae* while interactions between *C. decolorata* and its plant resources were constant with the null model.

**Conclusion:**

Our data are consistent with the hypothesis that behavioral differences in brood provisioning between solitary and social bees is a factor leading to relatively high variation in the microbiota of the solitary bee.

## Introduction

Interest for bee microbiota has recently been rising, alleviating the gap in knowledge in regard to drivers of solitary bee gut microbiota. However, no study has addressed the microbial acquisition routes of tropical solitary bees. For both social and solitary bees, the gut microbiota has several essential roles including biosynthesis of nutrients, degradation of pectin and lignocellulose, and dietary carbohydrate metabolism (Onchuru et al., 2018). These symbionts are also important for the host’s immune response to infections by pathogens, parasites, and parasitoids (Kwong et al., 2017; Onchuru et al., 2018; Steele et al., 2021). These critical immune roles have significant consequences for bee conservation (LeBuhn and Vargas Luna, 2021) as demonstrated by studies with the honeybee, *Apis mellifera*, the most important commercial honey producer and a highly valued species for the pollination services provided to crops (Hung et al., 2018). This social bee has been the most widely studied model organism in the field of bee gut microbiota. Regardless of the geography, environment, and subspecies, the microbiota of *A. mellifera* is highly conserved (Martinson et al., 2011), and is sometimes referred to as the global honeybee microbiome (Almeida et al., 2022). The composition of the honeybee core microbiota (a persistent set of low diversity bacterial phylotypes/OTUs) includes the following taxa: *Lactobacillus* Firm-4, *Lactobacillus* Firm-5, *Bifidobacterium*, *Gilliamella*, *Snodgrasella*, *Bartonella apis,* and *Frischella* and other Alphaproteobacteria (termed 2.1 group; Martinson et al., 2011; Kwong et al., 2017). The recurrence of the microbiota in these social bees results from (1) the transmission from the mother colony to daughter queens (vertical transmission), and (2) by social interactions between individuals of the same nest, including food exchange (trophallaxis; Michener, 1974). In other words, sociality plays an important role in the vertical transmission of the microbiota (Koch et al., 2013).

These low diversity and recurring phylotypes appear not only in honeybees but also bumblebees (Kwong et al., 2017) as well as other primitively social apids such as *Xylocopa* spp. (Handy et al., 2022; Holley et al., 2022). *Lactobacillus* Firm-4 and Firm-5 can also be found in low abundance in solitary bees (McFrederick et al., 2012, 2017; Graystock et al., 2017; Cohen et al., 2020). These trace levels could represent occasional horizontal transfers from social bees. Essentially, solitary bees do not share the core phylotypes of social bees and are still able to process food and respond to pathogens. The solitary bee microbiota seems to be species-specific with diverse bacteria likely playing similar roles of protection and nutrition. Indeed, these host-microbiota associations are important, as they contribute to the survival and the growth of larvae (Dharampal et al., 2019). How these larvae acquire their symbiotic bacteria and what role the mother bee plays in the microbial establishment remains underexplored.

Solitary *Centris decolorata* is an oil-collecting bee of the tribe Centridini, a sister clade to the corbiculates (Michener, 2000). It is a common bee species in coastal tropical environments (Alves-dos-Santos et al., 2009; Starr and Vélez, 2009), nesting in typical coastal vegetation. In Puerto Rico, they form large nest patches during the wet season (April to November; pers. obs.). Centridini is widely distributed and typically have high host plant species richness, large body sizes, and important interactions with many plant groups (Sigrist and Sazima, 2004; Gaglianone et al., 2010). They constitute the most ancient lineage of floral oil-collecting bees (Buchmann, 1987; Renner and Schaefer, 2010; Martins et al., 2014; Powell et al., 2014). Compared to large nests of honeybees (hives), the nests of *C. decolorata* are quite simple. They are constructed by individual females (mother bees) and consist of 15 cm-long tunnels dug diagonally into sandy soils, and generally have one brood cell per tunnel. The brood cell walls are composed of oils, leaf materials, resins and secretions from the Dufour’s glands (mostly aliphatic hydrocarbons; Roubik, 1989), which provide a hydrophobic barrier for the larva (Danforth et al., 2019). The source of oils is mainly from flowers of Malpighiaceae (Thiele and Inouye, 2007), which may be kilometers from the nest (pers. obs.). Oil collecting females provision each cell with pollen, mixed with oil, glandular secretions from Dufour’s glands, and an egg (Roubik, 1989; Danforth et al., 2019). The absence of evaporated nectar in brood cell provisions has yet to be chemically tested across a wider range of oil collecting bee species (Neff and Simpson, 2017). The completed brood cell has a coating or lining that confers humidity homeostasis, serving as the first-line defense to foreign microbes (Danforth et al., 2019), whereas the mixture of the provisions includes antimicrobials from mandibular gland secretions serving as the second-line defense (Cane et al., 1983).

Females sometimes forage far from the nest but always return to it, while males patrol the immediate vicinity of the nest without ever entering it. Where these nests occur along beaches in Puerto Rico, the vegetation typically consists of *Canavalia rosea, Ipomoea pes-caprae, Vigna luteola, Bidens Alba*, and *B. pilosa* (Martinez-Llaurador, 2021). At nest sites, territorial males form aggregation patches and exhibit perching behavior (Alves-dos-Santos et al., 2009; Starr and Vélez, 2009). The foraging niche of *C. decolorata* along coastal environments has been partially characterized by utilizing observation-based pollination networks (Martinez-Llaurador, 2021). Although such networks provide useful information on plant-pollinator relationships, some important interactions may be missed that a study of pollen load composition could provide (Forup and Memmott, 2005; Greenleaf et al., 2007; Jędrzejewska-Szmek and Zych, 2013; Fisogni et al., 2018). Characterizing pollen loads also offers a better understanding of how pollen use may influence microbial acquisition (Dew et al., 2020). In this study, we aim to characterize and compare the pollen load composition of *C. decolorata* and *A. mellifera* and relate it to microbiota diversity and composition. If there is no difference in pollen load composition between the two species yet their microbiota differ, then other acquisition routes may be involved, e.g., by soil, mother bee, or in this case other plant materials such as floral oils.

We asked whether the microbiota of a solitary bee in Puerto Rico is similar to that of co-occurring social *A. mellifera*, a variant known as “gentle Africanized honeybees” (*gAHB*). *Apis mellifera* also served as a positive control in the sense that its microbiota has been widely discussed and reported in the literature (*cf.* phylotypes cited above) as the global honeybee microbiome. Even though the honeybees of Puerto Rico are somewhat unique in having a mosaic of traits between European and Africanized honeybees (Rivera-Marchand et al., 2012), we expect that their microbiota should be similar to that reported in the literature since the global honeybee microbiome is consistent even across subspecies of *A. mellifera* (Almeida et al., 2022). These bees have a core gut microbiota that changes with developmental stages (Ortiz-Alvarado, 2019). Authors described a microbiota clustered into two well-defined groups: *Fructobacillus* genus (Phylum Firmicutes), Rhodospirillales and Acetobacteraceae (Phylum Proteobacteria) in early development stages, and Lactobacillaceae (Phylum Firmicutes), and Neisseriaceae (Phylum Proteobacteria) in late development stages (Ortiz-Alvarado, 2019).

As the solitary bee-microbiota is impacted by environmental acquisition routes (Voulgari-Kokota et al., 2019b), we expected higher microbial variation in *C. decolorata* compared to *A. mellifera.* We also hypothesized that more bacterial taxa would be shared between *C. decolorata* females and larvae, than that between males and larvae, due to female rearing and providing resources to the offspring. To our knowledge, this is the first study describing the differences in the gut microbiota between social and solitary bees in a tropical environment, while discussing the role of the solitary oil-collecting mother bees on the original gut microbiota of larvae.

## Materials and methods

### Bee collections and dissections

**Figure 1:**
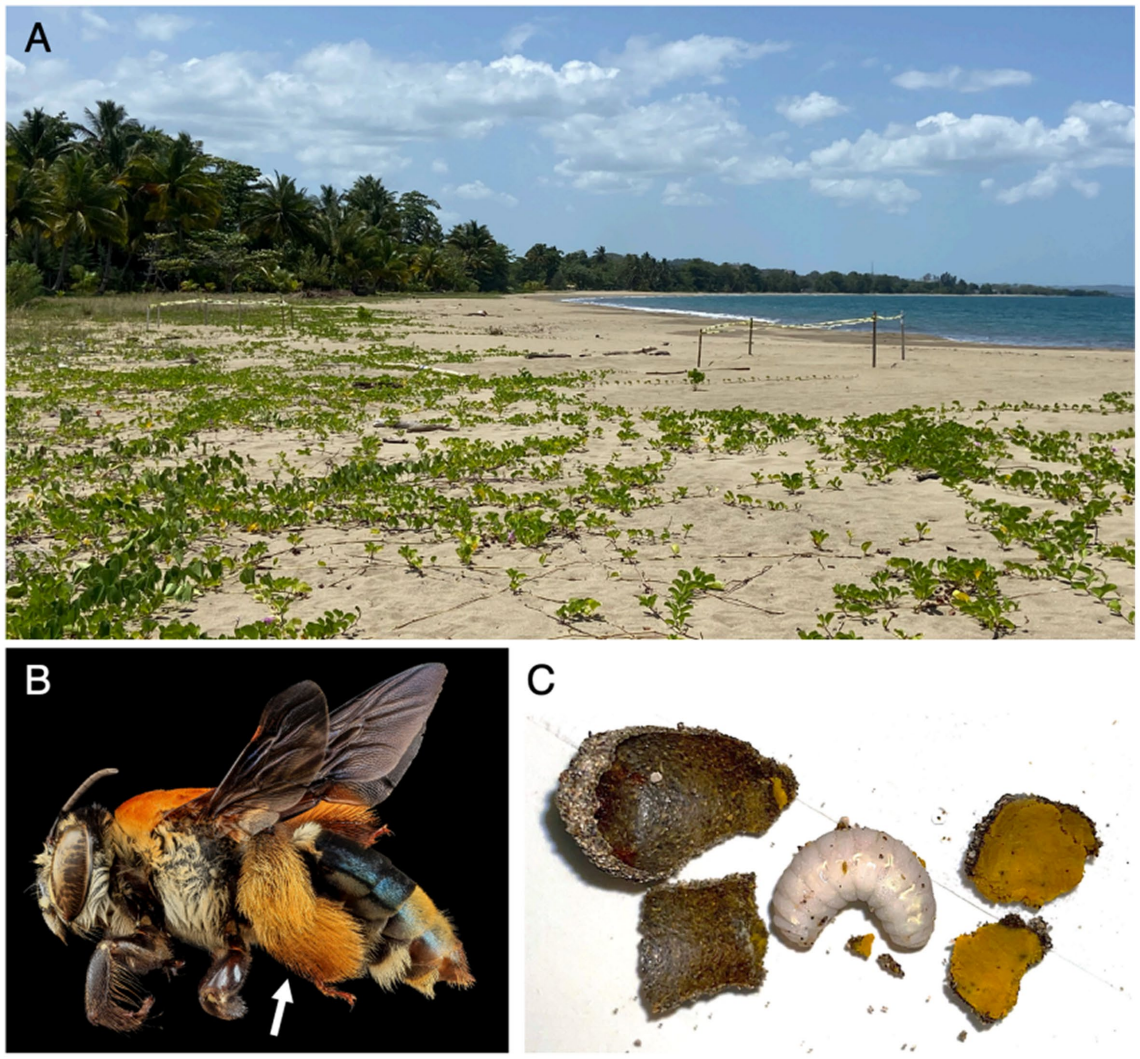
**(A)** Picture of the coastal environment where samples were collected. **(B)** Female *Centris decolorata*, arrow shows the hairy hindleg for pollen and floral oils collection. Credit: U.S. Geological Survey/photo by Wayne BooCanon. **(C)** Brood cell wall, larva of *C. decolorata* and brood cell provision.

On 15 May and 22 May 2022, *Apis mellifera* foragers and adult *Centris decolorata* were collected with an insect net (Departamento Recursos Naturales, permit ID 2022-IC-019). *Apis mellifera* (honeybees) were collected in three sites from two different towns, to make sure they came from different hives: Coamo (18.036814, −66.374096) and Mayagüez (2 plots, 18.250797, −67.177461 and 18.251412, −67.178063), Puerto Rico, United States. Mayagüez is a coastal town and in these exact coordinates, *Centris decolorata* specimens were also collected (Figure 1A). *Centris decolorata* nests were excavated in two Mayagüez plots following the method by Marinho et al. (2018). A total of 46 individuals were collected for this study. These individuals include, 9 *A. mellifera* foragers—6 collected from Coamo and 3 from Mayagüez–; and 24 *C. decolorata* bees (12 females, 12 males and 13 brood cell contents), all from Mayaguez (Figure 1B). The adult digestive tract (foregut to hindgut) of each species were dissected using sterilized tools under the stereomicroscope. The brood cells were also dissected to retrieve the whole individual larvae and the associated brood cell provision (Figure 1C). Because some brood cells were empty and solely contained the starting/remaining brood cell provisions and some *A. mellifera* had very small sizes and had to be pooled for extractions, a selection of 39 samples was done for analyses: 6 *A. mellifera* workers (female foragers); 10 female (mother bees) and 10 male *C. decolorata* adults, 4 of their larvae, and 10 of their brood cell provisions. Even though reproducing female solitary bees are further referred as “solitary mother bees,” their sampling has been done independently from their larvae. The female solitary bees we collected were considered as to be mother bees based on their behavior: returning to the nest at the end of the afternoon or carrying plant materials into the nest.

### Microbiota analysis

#### DNA extraction

The DNA of the adult guts and of the entire larval body was extracted using the PowerSoil Pro Kit (QIAGEN LLC, Germantown Road, Maryland, United States) following the manufacturer’s instructions, preceded by the addition of 20 μL of Proteinase K for 5 min. A Qubit® dsDNA HS assay kit (High Sensitivity; Waltham, Massachusetts, United States), was used to assess DNA concentrations of purified extracts (average DNA yield = 138 ng/μL).

The DNA obtained from all samples was normalized to 4 nM during 16S rRNA gene library preparation. We employed the Earth Microbiome Project standard protocols,^1^ using the universal bacterial primers: 515F (5′GTGCCAGCMGCCGCGGTAA3′) and 806R (5′GGACTACHVGGGTWTCTAAT3′) to amplify the hypervariable region V4 of the 16S ribosomal RNA gene (~291 bp) with region-specific primers that include sequencer adapter sequences used in the Illumina flowcell (Caporaso et al., 2012). Amplicons were quantified using PicoGreen (Invitrogen) and a plate reader (Infinite® 200 PRO, Tecan). Once quantified, volumes of each of the products were pooled into a single tube so that each amplicon is represented in equimolar amounts. This Pool is then cleaned up using AMPure XP Beads (Beckman Coulter), and then quantified using a fluorometer (Qubit, Invitrogen). Customized sequencing was outsourced at Argonne National Laboratory (Illinois, United States) using llumina MiSeq with the 2 × 250 bp paired-end sequencing kit. The reads obtained from the sequencer and its corresponding metadata were uploaded in QIITA study ID 14679. The raw data was made available at the European Nucleotide Archive Project (ENA) under the access number ERP141576.

#### Sequence processing and statistical analyses

The initial processing of the resulting Fastq files was done using QIITA (version 2022.07). This included demultiplexing and trimming to 200 bp, followed by deblurring against the SILVA database. Deblur methods to join, denoise, and duplicate sequences, including the removal of chimeric sequences, singleton reads, quality filtering, and joining of paired ends. The resulting *.biom* files (without taxonomy) were processed locally in QIIME2 (version 2022.02) and R (version 2021.09 build 351) after removing singleton reads and chloroplast/mitochondrial and plant related sequences. The bacterial sequences were classified using the pre-formatted SILVA 16S rRNA reference database and taxonomy files (138 release; Quast et al., 2012) trained with scikit-learn 0.24.1 (Pedregosa et al., 2012). The downstream processes with the *biom* table were followed as in previous studies (Rodríguez-Barreras et al., 2021; Ortiz et al., 2022; Ruiz Barrionuevo et al., 2022).

A set of microbiota analyses were done comparing (1) social and solitary bees (at their adult stage), and (2) solitary bees (adult males and females) and their brood cells (brood cell provisions and larvae), referred hereafter as “comparison group 1” and “comparison group 2.” For each comparison group, we computed analyses of beta and alpha diversity, taxonomic profiles, and putative biomarker taxa. Beta diversity analyses were done using the Bray–Curtis dissimilarity index and plotted using non-metric multidimensional scaling (NMDS) with samples colored according to the metadata categories, with 95% confidence ellipses. Beta diversity statistical tests including Permanova (Anderson, 2001), Permdisp (McArdle and Anderson, 2001), and Anosim (Clarke, 1993) were applied to quantify dissimilarity between both comparison groups. Permanova and Anosim were both applied to compare the dispersion of Bray–Curtis dissimilarity index in the Non-Metric Multidimensional Scaling. A Permdisp was used as an assumption of Permanova to test the null hypothesis of homogeneity of multivariate variances. For alpha diversity analyses, Chao 1 index (richness; Chao and Chiu, 2016) and Shannon (diversity index; Shannon, 1948) were visualized as boxplots using R (version 2021.09 build 351). Significant differences according to richness and diversity were assessed using Kruskal and Wallis (1952). Taxonomic profiles were visualized as standard QIIME2 barplots, and putative biomarker taxa differentially significant in multivariable associations with metadata variables were calculated in the package *maaslin* (Mallick et al., 2021). In addition, a core microbiota was identified for each variable of comparison group 2, using MicrobiomeAnalyst (Chong et al., 2020). The core microbiota considers taxa that are present in at least 50% of the samples for a given *C. decolorata* category (either female, male, larva, or brood cell provisions) and prevalence across samples for a given sample group is shown as heat colors.

### Pollen analysis

#### Pollen slide preparation

Pollen loads from *C. decolorata* and *A. mellifera* were stained with Calberla’s staining solution and analyzed with light microscopy. *Apis mellifera* legs as well as the body of *C. decolorata* were removed and placed over individual microscope slides (Wood et al., 2018). The contents of each microscope slide were bathed in 1–2 drops of ethyl acetate to wash off the pollen grains (Bezerra et al., 2020). Excess pollen grains still adhered to their legs and body were removed with the use of an entomological pin before staining with 2 drops of Calberla’s solution. Cover slip borders were sealed over each sample with clear nail Polish.

#### Pollen species identification

Pollen slides of *C. decolorata* and *A. mellifera* were observed in their entirety and pictures of the pollen grains were taken using an Olympus EP50 digital camera (Supplementary Figure 1). Pollen grains from each sample were counted manually, categorized based on their morphology and identified to the lowest taxonomic level possible, using available resources (PalDat, 2000; Halbritter et al., 2018). Pollen types that were not identified to the lowest taxonomic level were assigned a unique ID based on their morphological characteristics. In addition, a pollen reference catalog was created with pollen collected directly from plant species located at the study site.

#### Pollen statistical analysis

We compared pollen load composition between *C. decolorata* and *A. mellifera*, by using the Shannon diversity index and constructing a pollination network. Pollen grain types with a count of less than 5 grains were excluded from the analysis as they could have been accidentally collected or a result of contamination (Bosch et al., 2009; Fisogni et al., 2018). To calculate the proportion of the pollen volume of each pollen type, we measured the length of the polar and equatorial axes of 5 randomly encountered grains of each pollen type in each sample (da Silveira, 1991; O’Rourke and Buchmann, 1991; Stoner et al., 2022). Measurements were made using a calibrated EP50 digital camera at 400X. The volume of each pollen type was calculated using the average polar and equatorial lengths following the formulas for different shapes (O’Rourke and Buchmann, 1991). The proportion of the pollen volume of each pollen type was then calculated as follows:


$$\begin{array}{l} Pollen\;volume\;proportion\\ \;\;\;\;\;\;=\frac{Count\;of\;pollen\;grainsxVolume\;of\;pollen\;grains}{Sumof\;total\;volume\;forallpollen\;types\;in\;the\;sample} \end{array}$$

To account for the size and counts of each pollen type in each sample, the Shannon diversity index was calculated using the number of pollen grains multiplied by the volume. The interactions between *A. mellifera, C. decolorata,* and plant species were visualized using a pollination network plot based on the pollen volume proportion of pollen types found in individual samples. The network was constructed with the function “plot bipartite” of the package *econullnetr* (Vaughan et al., 2018). Plant resource selection was analyzed by running 1,000 simulations of null models. In addition, the function “plot preferences” of the *econullnetr* package was applied to better visualize and summarize the interaction strength between bee species and plant species. Plant species richness and diversity were compared using boxplots and a Kruskal-Wallis test was applied to assess differences between *C. decolorata* and *A. mellifera*. All indices and figures were produced using the R 4.2.2 version (PositTeam, 2022), and the *vegan* package was used to calculate plant diversity and richness (Oksanen et al., 2022).

## Results

After sequence filtration and rarefaction (rarefaction value of 1,045 reads per sample), only two of four samples of larvae yielded enough reads, suggesting a *nearly* sterile individual at early stages (for larval body length < 16 mm, 34 and 44 reads). These two low read samples of larvae were thus not included in the analysis. Because 25 nests had already been excavated to obtain 13 complete brood cells, including 4 with larvae, we decided not to excavate more solitary bee nests at this location for conservation reasons (Table 1). The microbial analysis including larvae (*n* = 2) is shown but should be considered with caution, given the very low sample size. Because minimum sample sizes for Kruskal-Wallis test is five, any analysis with less than that does not approximate the chi-square distribution accurately. Our best data in terms of sample size are *A. mellifera* foragers, female *C. decolorata* and brood cell provisions (Table 1).

**Table 1:** Summary of study variables, samples, reads and OTUs.

Species	Sample type	Details	*n*	Ave. reads	Ave. OTUs
*Apis mellifera*	Gut *A. mellifera*	Worker	6	17,995.17 ± 3543.57	47.5 ± 20.80
*Centris decolorata*	Gut *C. decolorata* female	Female	6	4,999.83 ± 3,807.87	42 ± 32.22
	Gut *C. decolorata* male	Male	4	2,673.75 ± 2824.71	80 ± 22.63
	larva	Larva	2	9,703.50 ± 7350.37	168.25 ± 112.15
	brood cell provision	pollen provision, and possibly nectar and oils	8	19,887.75 ± 3004.27	42.17 ± 10.23
Total			26	12583.62 ± 8083.15	85.08 ± 84.07

**Figure 2:**
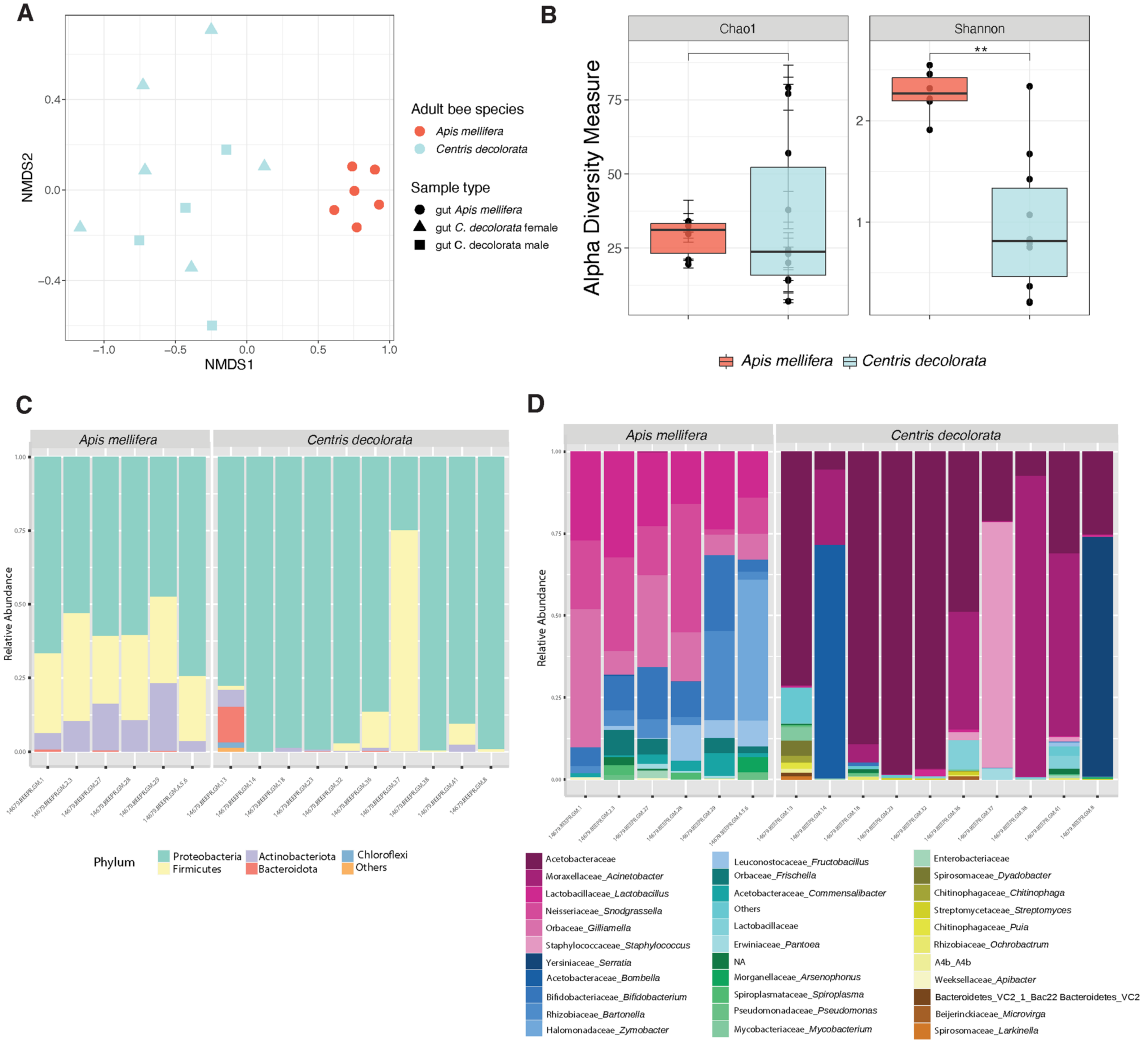
Diversity analyses comparing the microbiota of the two species of bees *Apis mellifera* (social) and *Centris decolorata* (solitary). **(A)** Beta diversity analysis, represented in a 2D NMDS with Bray-Curtis distances for species and sample types, depicts distinct clustering between the brood cell content and the adult bee with PERMANOVA value of *p* = 0.001; ANOSIM value of *p* = 0.001. **(B)** Alpha-diversity among species using Chao1 and Shannon indices. Asterisks depict significant values (*, **, *** representing 0.05, 0.01, 0.001, respectively). Bar Plots show the relative abundance (minimum 5%) of bacteria at the phyla **(C)**, and genus levels **(D).**

We found significant differences between the bacterial community structure of *A. mellifera* and *C. decolorata*. Beta diversity analyses revealed greater distances between *C. decolorata* individuals than between those of *A. mellifera* (PERMANOVA *p* = 0.001 and ANOSIM *p* = 0.001, Figure 2A; Supplementary Table 1). On the other hand, we found no difference in richness between *Apis mellifera* and *Centris decolorata* adults (Chao1 *p* = 0.914, Figure 2B; Supplementary Table 2). However, the gut microbiota of *A. mellifera* had a higher diversity than that of *C. decolorata* (Shannon *p* = 0.0048, Figure 2B; Supplementary Table 2).

### Core taxa in social vs. solitary bees

**Figure 3:**
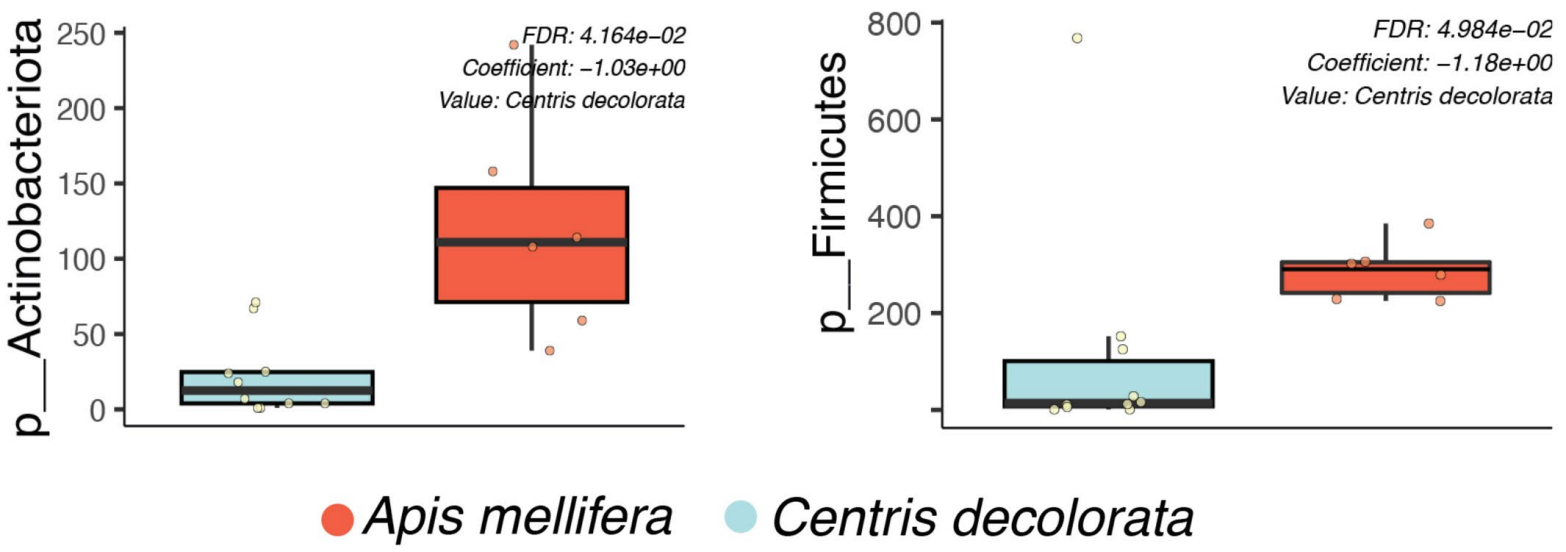
Bacterial phyla-level boxplots that discriminate among the two bee species with a q-value cut-off = 0.05. The corrected value of *p* for each taxon is shown in the upper right of the boxplots using MaAsLin.

**Figure 4:**
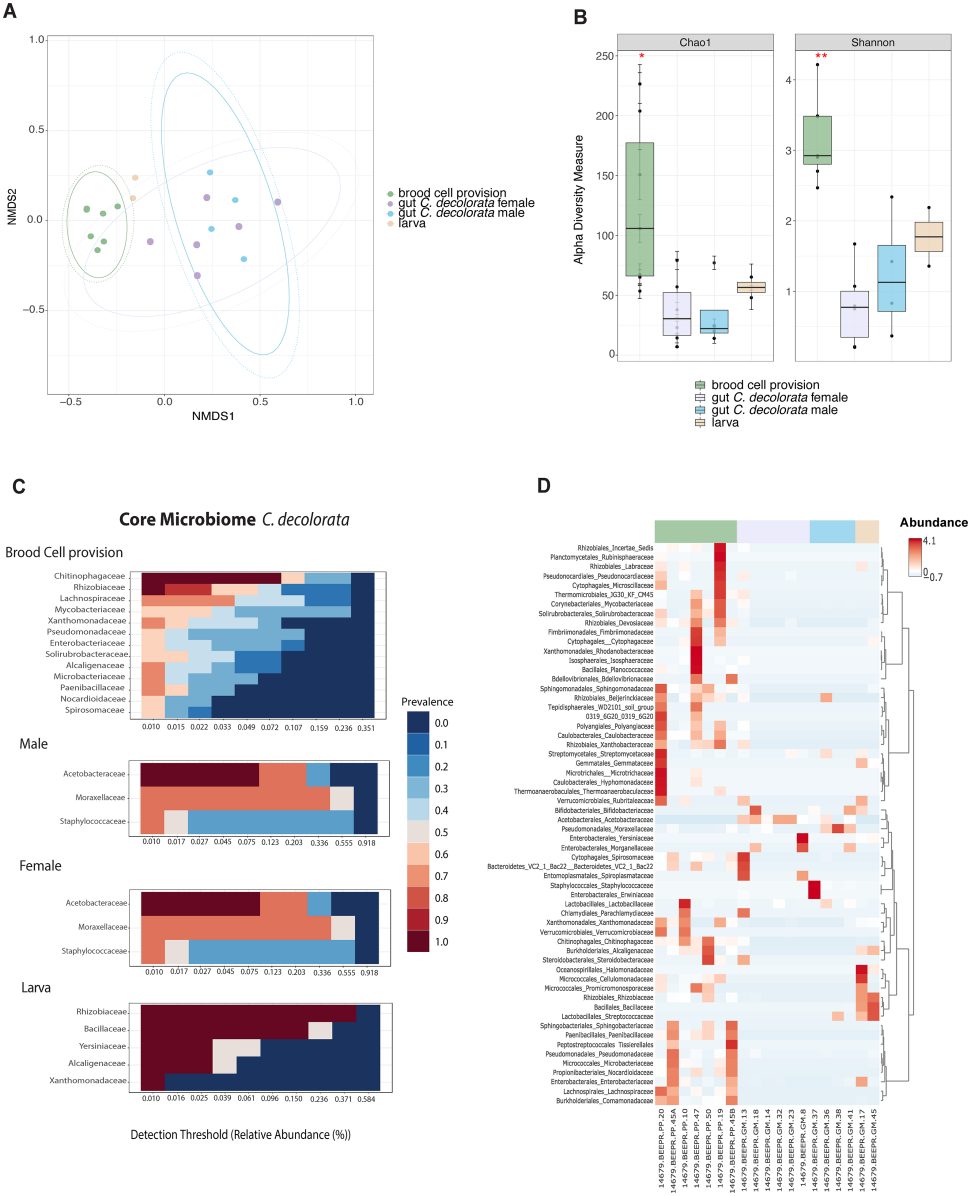
Overview of microbiota analyses for *Centris decolorata* samples (*n* = 19) considering larva and brood cell provisions. **(A)** Beta diversity analysis, represented in a 2D NMDS with Bray-Curtis distances for *Centris decolorata* samples, depicts distinct clustering between the brood cell content and the adult bee with PERMANOVA value of *p* = 0.001; ANOSIM value of *p* = 0.001. **(B)** Alpha-diversity among *C. decolorata* samples using Shannon index, stars are showing significative values, Shannon value of *p* between brood cell content and female = 0.001, and between brood cell content and male = 0.05. Heatmaps showing the relative abundance of the bacterial phylum-level (assigned per phylum) **(C)** Core bacterial biota at the family-level per each *C. decolorata* sample groupings, corresponding to taxa detected in a fraction of at least 50% of individuals with greater than 0.01% of relative abundance. Prevalence is show as heat colors **(D)** Taxonomic heatmap at the order and family-level for each sample groups.

In feral foragers of Puerto Rico honeybees, the simple and recurrent phylotypes of the gut microbiota remain as previously described in other honeybees (Martinson et al., 2011; Thompson et al., 2017). The gut microbiota of both bee species has the same phyla (Figures 2C, 3); however, the families are different. The core taxa of *Apis mellifera* comprises the families of Lactobacillaceae, Bifidobacteriaceae, Bartonellaceae, Neisseriaceae, Orbaceae, Rhizobiaceae, and Acetobacteraceae (i.e., *Commensalibacter* spp.; Figure 2D; Supplementary Figure 2). In contrast, the core microbiota of *Centris decolorata* is composed by bacteria from the Acetobacteraceae (i.e., undescribed Acetobacteraceae) and Moraxellaceae (Figures 2D, 4C,D). Some *C. decolorata* females displayed trace levels of undescribed species of Lactobacillaceae and Bifidobacteriaceae (Figure 2D; Supplementary Figure 2).

### The microbiota of brood cell provisions and adults of *Centris decolorata* are distinct

Microbiota composition and structure of brood cell provisions are different from all other samples (Figures 4A,B). Brood cell provisions had significantly higher diversity than any gut microbiota of adult solitary bees, but not strongly different than larvae (alpha-diversity differences using Chao1, adjusted-*p* = 0.286 and using Shannon, adjusted-*p* = 0.081; Supplementary Table 1). While no significant differences in diversity metrics were found between larvae and brood cell provisions, both beta and alpha diversities of brood cell provisions are significantly different from that of adult females and males (PERMANOVA, *p* = 0.001; ANOSIM, *p* = 0.001; Chao1 and Shannon index, *p* < 0.05, Supplementary Tables 1, 2; Figures 4A,B). Brood cell provisions are composed by diverse families of bacteria having a higher number of taxa as part of the core microbiome as compared to other sample types. Females and males were mostly composed in Acetobacteraceae and in Moraxellaceae (Figure 4C), in fact only Acetobacteraceae are part of the female core microbiome. Furthermore, brood cell contents and adults did not display the same core microbiota at 50% sample prevalence. Rhizobiaceae, Chitinophagaceae, Lachnospiraceae, Enterobacteriaceae, Xanthomonadaceae, Alcaligenaceae, and Microbacteriaceae constituted the core microbiota of brood cells, while Acetobacteraceae constituted that of adults (Figures 4C,D). Undescribed Acetobacteraceae explained most of the differences between adults and brood cells (mostly females) using MaAsLin statistical analysis. Only males had abundant *Staphylococcus* sp. (Supplementary Figure 3).

### Pollen diet in *Apis mellifera* and *Centris decolorata*

A total of 28 pollen types were identified from *A. mellifera* and *C. decolorata* pollen loads (*cf.* “Plant Pollen and Bee Pollen Grain Catalog” in Supplementary Presentations 1, 2). Of these, 16 pollen types were excluded after filtering for pollen types with less than five grains per slide. Seven of the remaining pollen types were found in *C. decolorata*. Three pollen types, including *Byrsonima* sp. (an oil flower) and *Combretaceae*, were found shared by both bee species (Supplementary Figure 4A). The pollen types associated with *C. decolorata* did not reflect the plant species near their nests, such as *Canavalia rosea*, *Ipomoea pes-caprae,* and *Bidens Alba* (Martinez-Llaurador, 2021). *Apis mellifera* had a weaker interaction with *Combretaceae* sp.1 and a stronger interaction with *Byrsonima* sp. than expected compared to the null model (Supplementary Figure 4B). The remaining interactions between both bee species and plant resources were described as consistent with the null model (Supplementary Figures 4B,C). Although *A. mellifera* had a higher plant resource species richness and diversity than *C. decolorata*, there were no significant differences (Kruskal-Wallis test *p* = 0.20 and *p* = 0.28 respectively).

## Discussion

Our first attempt to compare the gut microbiota of social (*A. mellifera*) and solitary (*C. decolorata*) bees has revealed that (1) microbial variability is higher in *C. decolorata* compared to *A. mellifera* and (2) for the solitary bee, the microbiota of their brood cell contents is significantly different from the gut microbiota of adults.

### Life history influences the gut microbiota of bees, as well as their nest microbiota

The lower physical contact between solitary bee individuals, compared to social ones (Wittwer et al., 2017) is one of the factors that lead to variability in microbial communities among individuals. With social interactions, including trophallaxis, social bees directly share their gut bacteria, reducing probability of interindividual variation. This participates in the maintenance of a consistent core gut microbiota. Compared to social bees, environmental transmission pathways of solitary bees play a stronger role in the acquisition of bacteria, probably due to differences in nesting habits and materials (Voulgari-Kokota et al., 2019b). Solitary bees such as *Centris* use pollen, nectar, secretions from mandibular and Dufour’s glands, and floral oils to build their nest. After the brood cell is completed, provisioned, provided with an egg, and sealed, the female has no contact with its brood. Through various strategies, the brood is protected from parasites, microbes, predators, and external environment variation, which is especially important in warm and humid environments (Danforth et al., 2019). As a first-line defense, female solitary bees coat their brood cells with glandular secretions which may be combined with other collected materials. Secretion from their Dufour’s gland is the primary source for lining brood cells. It consists mostly of large polar molecules, providing waxy, hydrophobic coating to the brood cell (Danforth et al., 2019). Their exact composition varies among *Centris* species (Cane and Brooks, 1983), though *Centris* from the Antilles have yet to be analyzed. Further studies should evaluate if female *Centris* use these secretions only to coat the brood cells or also to mix them with provisions, as do some other solitary bees, e.g., Megachilids (Williams et al., 1986).

Some solitary bees also use mandibular gland secretions as antimicrobials (Cane et al., 1983), sometimes to first disinfect the brood cell prior to lining (Cane and Tengö, 1981). For instance, linalool, citral, geraniol, nerol or citronellol, all mandibular secretions, are effective inhibitors of fungal and bacterial growth in multiple species of solitary bees (Cane et al., 1983). These molecules and their specific targets are yet to be described for Centridini. Floral oils may also serve as protective coating materials (mostly stearic acid and elaiophore lipids; Danforth et al., 2019) which females collect from multiple plant families such as Malpighiaceae, Calceolariaceae, Iridaceae, Orchidaceae, Krameriaceae, Plantaginaceae, and Solanaceae (Martins et al., 2015).

In addition to brood cell lining, females may also mix the floral oils with provisions as an energy source (Buchmann, 1987), but the generality of this incorporation is not well understood, due to the paucity of species for which nest provisions and linings have been chemically analyzed (Neff and Simpson, 2017). Whether floral oils replace nectar is not absolute: brood cell provisions may contain trace to appreciable amounts of sugar and oils (Neff and Simpson, 1981a). Alternatively, Neff and Simpson proposed that mixing floral oils with provisions is advantageous to oil-collecting bees nesting in environments susceptible to flooding. This argument is based on the absence of oil-collecting habits for *Centris* species in xeric habitats. Indeed, incorporating floral oils to the provisioning could inhibit hygroscopic effects of provisions from bees nesting in extremely moist environments, but also control mold or bacterial infection, or act as a deterrent against nest parasites (Neff and Simpson, 1981b). For instance, levulinic acid, an oil collected for brood cells, acts as an anti-fungal agent (Neff and Simpson, 1981a).

Given this wide variety of materials used for brood cell construction and provisioning, it is not surprising to see such diverse microbiota present in the guts of adult solitary bees, and even more in the brood cell provisions. How these bacteria survive in this antimicrobial, yet nutrient-rich brood cell microcosm is unknown, yet. We assume that the presence of these bacteria is important, as they will determine the digestibility of the raw pollen clump by larvae. Further research should describe the chemical composition of the brood cell provisions of Centridini bees, as well as the microbial targets of glandular secretions and floral oils.

### High microbial diversity in brood cells related to mass provisioning by mother solitary bees

While other studies showed that bacteria from the honeybee gut is transferred to their corbicula pollen during the process of pollen packing (Prado et al., 2022), the bacteria isolated from brood cells of *C. decolorata* clearly have a plant origin. Some of these bacteria are known to induce plant growth, e.g., Rhizobiaceae, Chitinophagaceae, or Lachnospiraceae, or to inhibit it, e.g., Xanthomonadaceae, or Alcaligenaceae (Gnanamanickam, 2006). It would be interesting to test if bees are able to modulate the abundance of plant-inhibiting bacteria in later stages of brood cell provisions, as pollen from brood cells is no longer available for pollination. Another constituent of the core microbiome from *C. decolorata*’s brood cell, Enterobacteriaceae, has been previously found in pollen (Madmony et al., 2005; Ambika Manirajan et al., 2016; Straumite et al., 2022) and larvae (Parmentier et al., 2018). This latter family also has a plant origin, especially flowers (Gnanamanickam, 2006; Junker et al., 2011; Junker and Keller, 2015). Other studies reported on the presence of some *Lactobacillus*, namely *L. micheneri*, *L. timberlakei*, and *L. quenuiae*, in the pollen provisions, bee guts and flowers. In addition to being tolerant of osmotic stress, these lactic acid bacteria are able to degrade the outer pollen wall (Lipiński, 2018), which makes pollen digestible for early larval feeding (Gurevitch and Padilla, 2004; Gilliam, 2006; Vuong et al., 2019; Voulgari-Kokota et al., 2019a). Given *Lactobacillus* presence in trace amounts, future description of the brood cell core microbiome combined to pollen wall degradation analyses at brood cell age should help identifying bacteria involved in pollen pre-digestion process.

All these brood cell constituents (pollen, possibly evaporated nectar and floral oils, and the associated bacteria) are definitely brought by *Centris decolorata* females to the larvae. But even though females are the ones provisioning the brood cells for the larvae, their gut microbiota is significantly different from the microbiota of the brood cell provisions, at least at early stages. These results contrast with previous findings of another solitary bee from a semi-arid region (although in dense aggregations of millions of bees), where the gut microbiota of females and larvae were similar (Kapheim et al., 2021). In *C. decolorata*, females provision the brood cell for the larva independently to what she ingests. In our study, the gut microbiota of both male and female solitary bees are more similar to each other than to that of brood cell contents, at least initially. Indeed, the collected brood cells were at early stages of larval development. The provisions were thus essentially composed in flower-specific bacteria, which is coherent considering that provisions are mainly constituted by pollen. Later, these flower-specific bacteria shift to bacteria able to grow on nutrient rich mixture, i.e., the proteins from pollen, and possibly sugar from nectar and floral oils. Microbial composition of the larvae and provisions therefore changes along with larval development (Voulgari-Kokota et al., 2018). To confirm the differences in microbial composition between females and nest provisions, future microbial assessment should consider a larger brood cell sampling, with early and later larval stages (using larval development as a proxy for bacterial shift). Indeed, assessing the brood cell provisions at the middle/end of the *C. decolorata* season would probably lead to higher probability to encounter brood cells of later larval stages. These brood cells would thus be composed in bacteria able to grow on nutrient rich mixture, probably similar to microbiota of females and larvae.

### Dominance of acetic acid bacteria in nesting solitary bees

Gut acidification of solitary and social bees seems to be driven by different phylotypes: Lactic Acid Bacteria in honeybees vs. Acetic Acid Bacteria in solitary *C. decolorata.* The trace amounts of Lactobacillaceae in *C. decolorata* guts could represent horizontal acquisition from *A. mellifera*, or environmental pools of related strains to *C. decolorata*. Acetic Acid Bacteria (AAB) are strict aerobic bacteria and ubiquitous. They occur in a wide variety of substrates such as in plants and flowers (Crotti et al., 2010). They are widespread in carbohydrate-rich, acidic, and alcoholic niches, such as nectar, which has been proposed as an origin for these bacteria (Morris et al., 2019; Ravenscraft et al., 2019). In addition to being considered environmental and ubiquitous bacteria, AAB are also important insect symbionts, as for food uptake and host survival. Insect associations are stable and follow several transmission routes for their propagation (Crotti et al., 2010). In our samples, two families of AAB were found: Moraxellaceae and Acetobacteraceae. Bacteria from the Moraxellaceae family, especially *Acinetobacter* have been isolated from the solitary male guts, but not from the brood cell provisions of our study, even though reported in the literature as present in pollen provisions coming from Mediterranean plants (Álvarez-Pérez et al., 2013). Although not found in pollen provision of our study, the bacteria from Acetobacteraceae were present in the guts of adult *Centris decolorata* (both males and females) and constitute their core microbiome. It was scarcely present in foraging honeybees that typically do not interact with larvae in hives (Winston, 1987), as reported in domestic local honeybees (gentle Africanized Honeybee, *gAHB*) by Ortiz-Alvarado (2019). Acetobacteraceae has been isolated from the gut of adult honeybees (Sabree et al., 2012; Anderson et al., 2013; Corby-Harris et al., 2014; Thompson et al., 2017; Ortiz-Alvarado, 2019), and different Acetobacteraceae (i.e., Alpha 2.2 Acetobacteraceae) from honeybee larvae (Corby-Harris et al., 2014; Ortiz-Alvarado, 2019). Interestingly, Acetobacteraceae was found in larvae, nymphs, young nest bees, and royal jelly in the same study, but it was almost absent in honeybee foragers. Acetobacteraceae could thus be a family of bacteria related to nursing bees, i.e., larvae, nymphs, young nest bees, and royal jelly in honeybees, and in female solitary bees, who play a nursing role. The absence of Acetobacteraceae in the brood cell of this study could suggest that the transfer of these symbionts could mostly be vertical for *Centris decolorata,* but this remains to be identified in a larger sample size of solitary bee larvae. If AAB such as Acetobacteraceae are present in females and larvae but not in the brood cell provisions, then vertical transmission of these symbionts would be preferred by *Centris decolorata*.

### Pollen acquisition routes do not explain microbial differences between solitary and social bees

Some Megachilids show a significant association between the composition of their foraged pollen and the pollen bacterial communities and larval bacterial communities. In these bees, where bacterial transmission pathways through eusociality are impossible, pollen foraging appears to be very important to obtain their bacterial symbionts (Voulgari-Kokota et al., 2019a). In our limited pollen study, we found that pollen resources foraged by *Apis mellifera* and *Centris decolorata* were not significantly different whereas their gut microbiota were composed of different bacterial phylotypes. Therefore, pollen acquisition routes cannot explain differences in the gut microbiota between the studied social and solitary bees. *Apis mellifera* has been previously shown to be weakly impacted by microbiota from pollen (Donkersley et al., 2018; Jones et al., 2018). Pollen samples of *C. decolorata* evidence the presence of additional plant resources along their foraging range, suggesting they forage for pollen over long distances from their nest locations, in addition to use pollen resources from plant species found near their nests (Pers. Obs.). Interactions between *C. decolorata* and *Byrsonima* sp. reflected a lower pollen abundance, suggesting that individuals visit *Byrsonima* sp. to primarily collect oils and incidentally collect pollen along their bodies. Although *C. decolorata* transports fewer pollen grains between individuals of *Byrsonima* sp*.,* these could be sufficient to pollinate the flowers. However, *A. mellifera* appears to be an effective pollen forager having a stronger interaction, influenced by a greater abundance of pollen on its corbicula, with *Byrsonima* sp. For a broader overview of plant species visited by *C. decolorata*, bee sampling and pollen analyses over the season and on different daily periods should be considered for future studies.

## Conclusion

Bee population decline is a global threat with possible losses of important ecosystem services which they provide, most importantly pollination. While most bee species are solitary, these have been understudied compared to social bees (e.g., honeybees and bumblebees). Unfortunately, conservation strategies to reverse population declines may not be the same for solitary bees as they may be for social bees. To our knowledge, the present study is the first microbiota inventory from a tropical solitary bee. We collected solitary bees in Puerto Rico and characterized the gut microbiota in adults and brood cells. A higher microbial variability in *Centris decolorata* was observed compared to co-occurring, feral *Apis mellifera*, and unexpectedly there was a low number of shared bacteria between females and brood cell contents. Even though female solitary bees are the ones rearing and providing resources to the offspring, larvae and their brood cell provision differ significantly from adult males and females. Females thus provide an independent provisioning of materials to the brood cells affecting their microbiota. These results highlight diversity in wild solitary bees, i.e., remarkable diversity in morphological traits, nesting habits and host-plant associations (Danforth et al., 2019), and their differences from wild social bees, e.g., *Bombus terrestris* which has relatively long period of activity, a tolerance for temperate extremes, and a broad diet (Ghisbain, 2021). As such, this study points to the need for further research on microbiota, pollen sources, and metabolism of this and other solitary bees for developing conservation strategies and securing pollination services. The coastal oil-collecting bee *Centris decolorata* is indeed an important ecosystem service provider, as it nests in the dunes and pollinates its vegetation. Indirectly, pollination by *C. decolorata* acts as a barrier to erosion, especially in case of extreme climatic events such as hurricanes.

## Data availability statement

The datasets presented in this study can be found in online repositories. The names of the repository/repositories and accession number(s) can be found at: https://www.ebi.ac.uk/ena, ERP141576.

## Author contributions

EK and FG-V: conceptualization. EK: data curation. EK, AG-R, and FG-V: formal analysis. FG-V: funding acquisition, project administration, and supervision. EK, FG-V, AG-R, and JA: investigation and writing—original draft. EK, AG-R, and JA: methodology. EK, AG-R, TG, JA, and FG-V: writing—review and editing. All authors contributed to the article and approved the submitted version.

## Funding

This work was supported by the Puerto Rico IDeA Networks of Biomedical Research Excellence, Advancing Competitive Biomedical Research in Puerto Rico, 5P20GM103475-20, and Center for Collaborative Research in Minority Health and Health Disparities (RCMI), 2U54MD007600.

## Conflict of interest

The authors declare that the research was conducted in the absence of any commercial or financial relationships that could be construed as a potential conflict of interest.

## Publisher’s note

All claims expressed in this article are solely those of the authors and do not necessarily represent those of their affiliated organizations, or those of the publisher, the editors and the reviewers. Any product that may be evaluated in this article, or claim that may be made by its manufacturer, is not guaranteed or endorsed by the publisher.

## Footnotes

^1^ https://earthmicrobiome.org/protocols-and-standards/16s/
