# Supplementary material for: Gut microbiota variation of a tropical oil-collecting bee species far exceeds that of the honeybee
Source: Front Microbiol. 2023 May 17;14:1122489. doi: 10.3389/fmicb.2023.1122489 (PMC10229882; doi:10.3389/fmicb.2023.1122489)
Supplement: Supplementary file 1 [file Image_1.pdf]

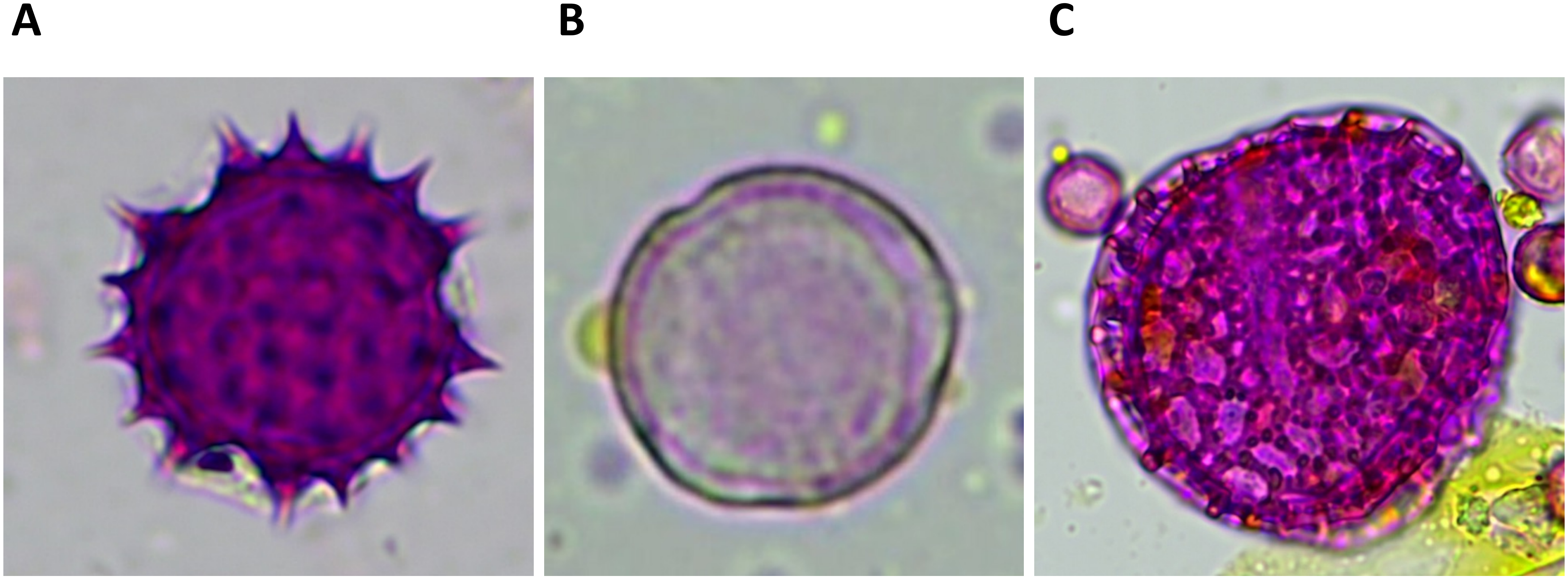


**Supplementary Figure 1. Examples of pollen grain pictures** of three plant families commonly observed (400x) in samples of *A. mellifera* and *C. decolorata*. **(A)** Asteraceae, **(B)** Malpighiaceae, and **(C)** a large Passiflora pollen grain surrounded by pollen grains of *Byrsonima sp.*
