# Supplementary material for: Gut microbiota variation of a tropical oil-collecting bee species far exceeds that of the honeybee
Source: Front Microbiol. 2023 May 17;14:1122489. doi: 10.3389/fmicb.2023.1122489 (PMC10229882; doi:10.3389/fmicb.2023.1122489)
Supplement: Supplementary file 2 [file Image_2.pdf]

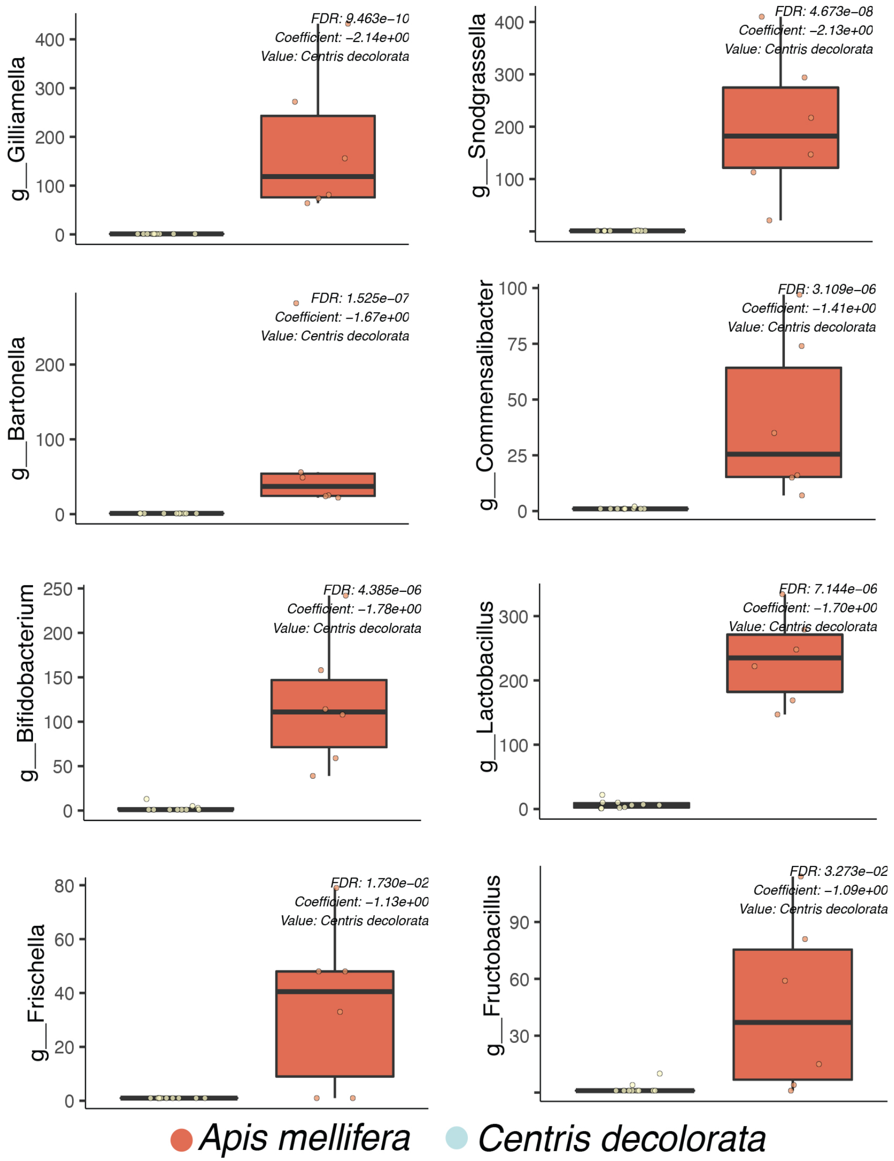

Supplementary Figure 2. Boxplots of bacterial genus-level (assigned per genus) that discriminate among the two bee species with a q-value cut-off =0.05, the corrected p-value for each taxon is shown in the upper right of the boxplots using MaAsLin.
