# Supplementary material for: Gut microbiota variation of a tropical oil-collecting bee species far exceeds that of the honeybee
Source: Front Microbiol. 2023 May 17;14:1122489. doi: 10.3389/fmicb.2023.1122489 (PMC10229882; doi:10.3389/fmicb.2023.1122489)
Supplement: Supplementary file 3 [file Image_3.pdf]

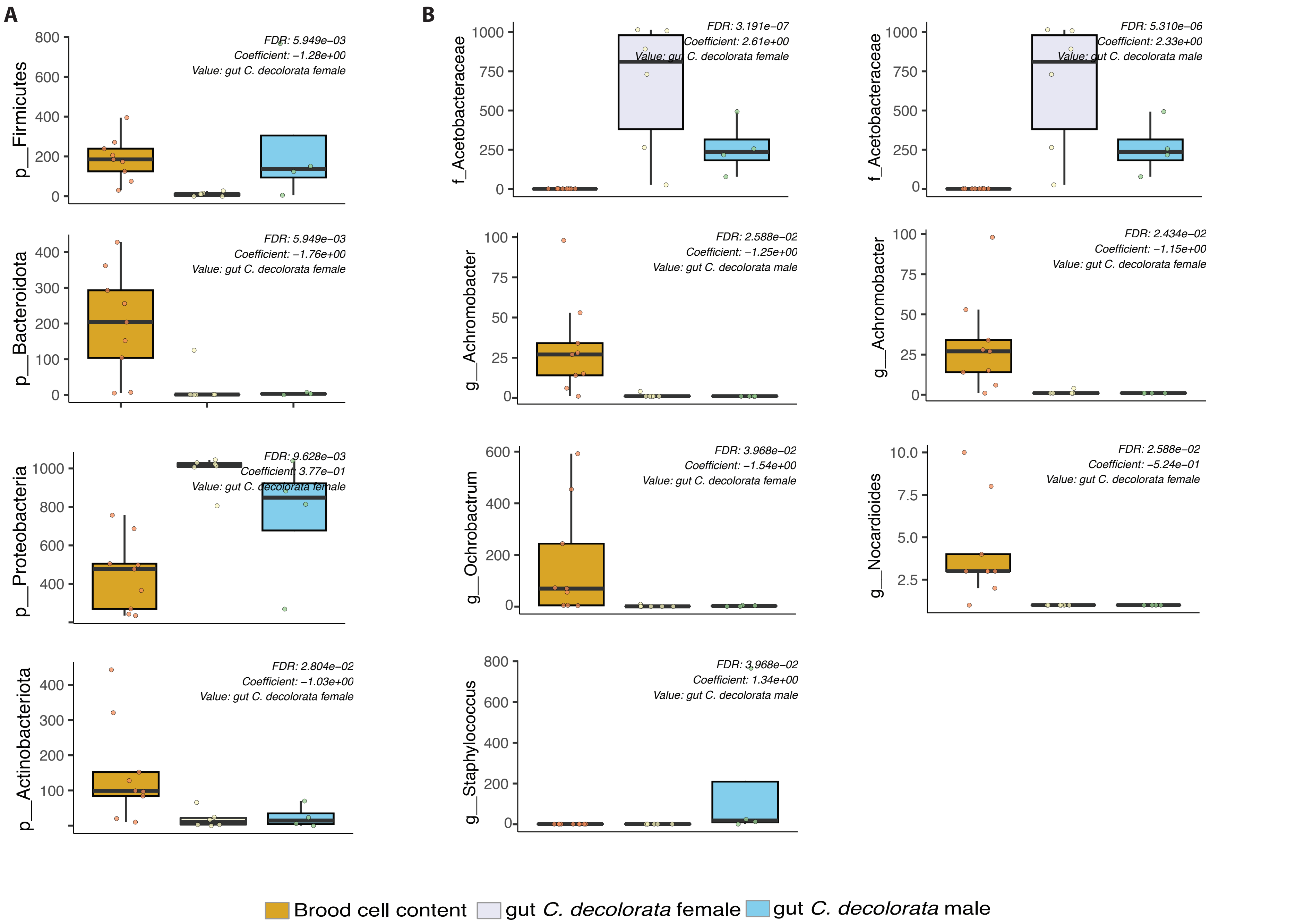

Supplementary Figure 3. Boxplots of bacterial phylum-level (assigned per phylum) (A) and genus-level (assigned per genus) (B) that discriminate among the C. decolorata samples with a q-value cut-off =0.05, the corrected p-value for each taxon is shown in the upper right of the boxplots using MaAsLin.
