# Supplementary material for: Gut microbiota variation of a tropical oil-collecting bee species far exceeds that of the honeybee
Source: Front Microbiol. 2023 May 17;14:1122489. doi: 10.3389/fmicb.2023.1122489 (PMC10229882; doi:10.3389/fmicb.2023.1122489)
Supplement: Supplementary file 4 [file Image_4.pdf]

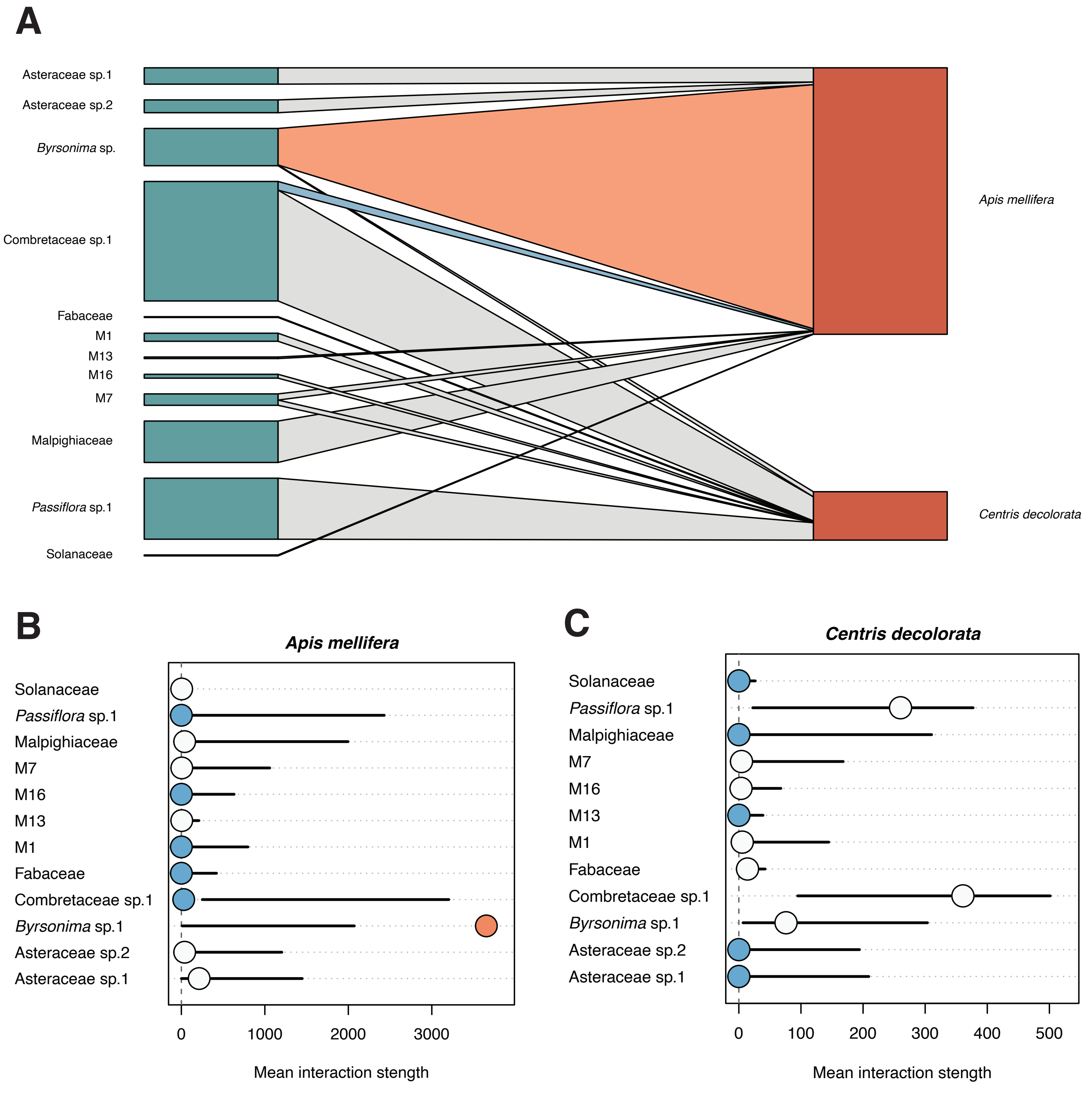

Supplementary Figure 4. **(A)** Pollination network of *Apis mellifera* and *Centris decolorata*. The labels containing the letter 'M' represent pollen types that were classified by their morphological characteristics. Based on the number of pollen grains and their volume, *A. mellifera* and *C. decolorata* did not present significant differences in plant species richness and diversity (Kruskal-Wallis test $p = 0.20$ and $p= 0.28$, respectively). The interaction width is influenced by pollen type abundance corrected by their volume for all samples. Interactions are color-coded according to whether they are consistent with the null model (grey), stronger (red), or weaker (blue) than expected. Both *A. mellifera* and *C. decolorata* had a weaker interaction with Combretaceae sp.1 compared to the null model. *A. mellifera* presented a stronger interaction with *Byrsonima* sp.1, while *C. decolorata* had a stronger interaction with *Passiflora* sp.1 than expected. Dot charts present observed and expected interaction strengths for **(B)** *A. mellifera*, **(C)** *C. decolorata*, and their plant resources.
