# Supplementary material for: Gut microbiota variation of a tropical oil-collecting bee species far exceeds that of the honeybee
Source: Front Microbiol. 2023 May 17;14:1122489. doi: 10.3389/fmicb.2023.1122489 (PMC10229882; doi:10.3389/fmicb.2023.1122489)

Catalog of pollen from common plant species found on Puerto Rico's coasts

Note 1: Each pollen type listed below has a 'Plant ID' that corresponds to the ID given to the pollen slide created for each plant species collected on the coast of Humacao, and a description of its morphology based on visible characteristics of the pollen grain. The collection of pollen slides is located in the laboratory of the Museum of Zoology at the UPR-RP. Pollen grains were observed using light microscopy at 400X and pictures were taken using an EP50 camera. Lastly, descriptions were assigned using the *Pollen Illustrated Terminology* by Halbritter et al. (2018).

Note 2: Images of the plant pollen slides and of the bee samples are available upon request.

1. Plant ID: P1

Coccoloba uvifera

Description:

a. **Pollen unit:** Monad
b. **Size:** Small
c. **Polarity:** Isopolar
d. **Shape:** Spheroidal
e. **Aperture type:**
f. **Aperture condition:** Tricolporate
g. **Ornamentation:** Verrucate

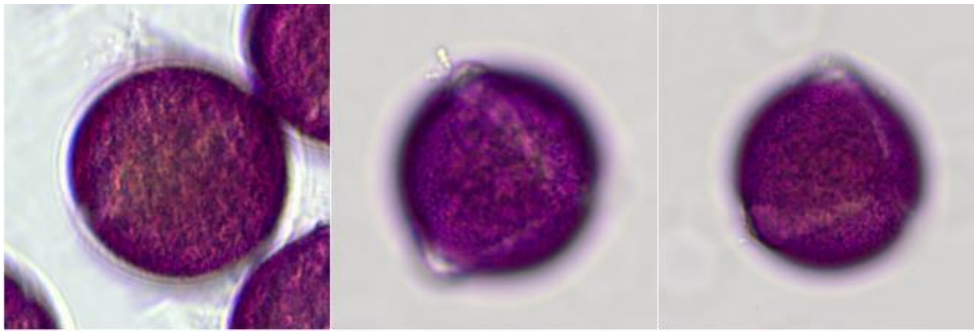

2. Plant ID: P2

Phyla nodiflora

Description:

a. **Pollen unit:** Monad
b. **Size:** Small
c. **Polarity:** Isopolar
d. **Shape:** Spheroidal
e. **Aperture type:**
f. **Aperture condition:** Tricolporate, coporate
g. **Ornamentation:** NA

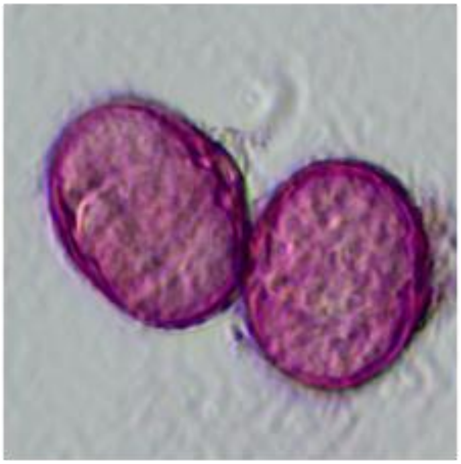

3. Plant ID: P3

Sphagneticola trilobata

Description:

a. **Pollen unit:** Monad
b. **Size:** Large
c. **Polarity:** Isopolar
d. **Shape: Spheroidal**
e. **Aperture type:** Colporus
f. **Aperture condition:** Colporate
g. **Ornamentation:** Echinate

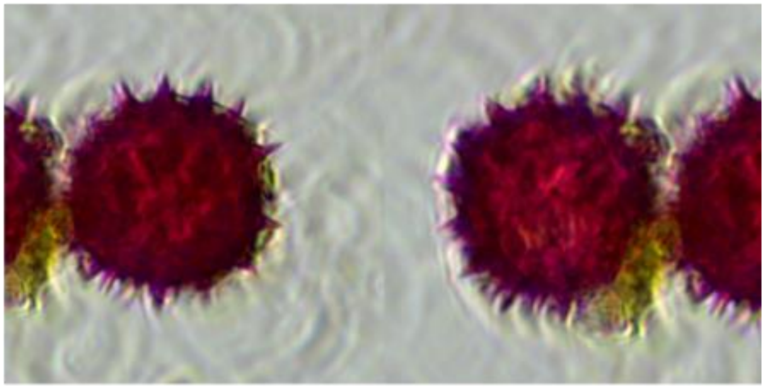

4. Plant ID: P4

Ipomoea pes-caprae (Pink/Violet)

Description:

a. **Pollen unit:** Monad
b. **Size:** Large
c. **Polarity:** Isopolar
d. **Shape:** Spheroidal
e. **Aperture type:** Colporus
f. **Aperture condition:** Colporate
g. **Ornamentation:** Echinate

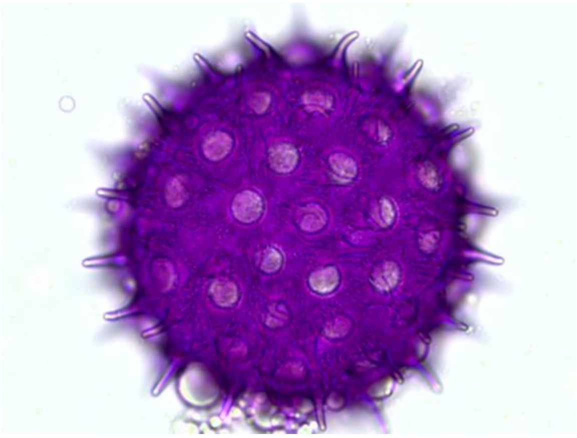

5. Plant ID: P6

Canavalia rosea

Description:

a. **Pollen unit:** Monad
b. **Size:** Small
c. **Polarity:** Isopolar
d. **Shape:** Triangular
e. **Aperture type:**
f. **Aperture condition:** Tricolporate, coporate
g. **Ornamentation:** NA

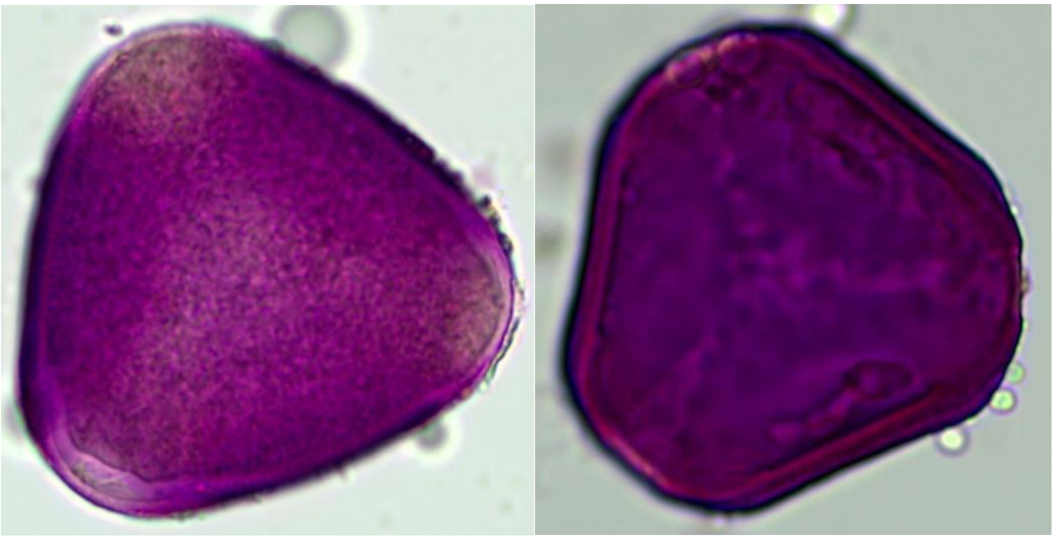

6. Plant ID: P7

Ipomea imperati

Description:

a. **Pollen unit:** Monad
b. **Size:** Large
c. **Polarity:** Isopolar
d. **Shape:** Spheroidal
e. **Aperture type:** Colporus
f. **Aperture condition:** Colporate
g. **Ornamentation:** Echinate

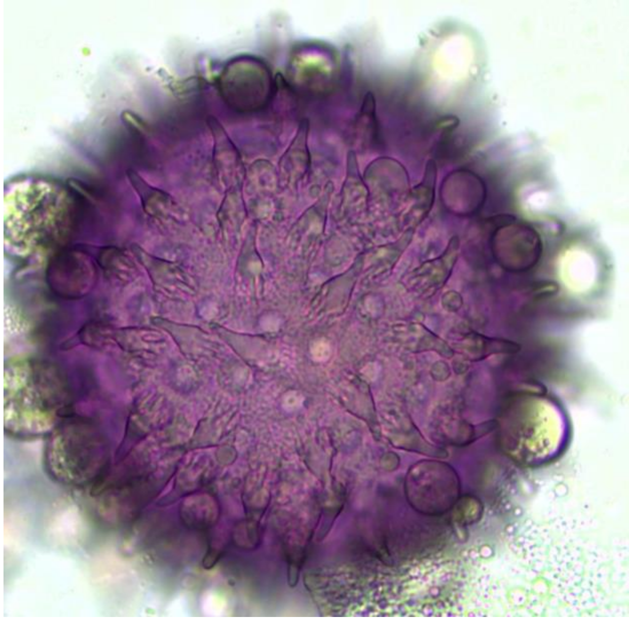

Supplement: Supplementary file 5 [file Presentation_1.pdf]
