# Supplementary material for: Gut microbiota variation of a tropical oil-collecting bee species far exceeds that of the honeybee
Source: Front Microbiol. 2023 May 17;14:1122489. doi: 10.3389/fmicb.2023.1122489 (PMC10229882; doi:10.3389/fmicb.2023.1122489)

Catalog of different pollen types found in samples of *A. mellifera* and *C. decolorata*

Note 1: Each pollen type has a label that corresponds to its lowest taxonomic level or morphotype, and contains a description of its morphology based on visible characteristics of the pollen grain. The letter 'M' stands for morphotype for those pollen grains that still haven't been identified to the lowest taxonomic level. Pollen grains were observed using light microscopy at 400X and pictures were taken using an EP50 camera. Lastly, descriptions were assigned using the *Pollen Illustrated Terminology* by Halbritter et al. (2018).

Note 2: Images of the plant pollen slides and of the bee samples are available upon request.

1. Label: *Byrsonima* sp.

Description:

a. **Pollen unit:** Monad
b. **Size:** Small (8-12 μm)
c. **Polarity:** Isopolar
d. **Shape:** Spheroidal
e. **Aperture type:** Colporus
f. **Aperture condition:** Tricolporate
g. **Ornamentation:** Verrucate, scabrate

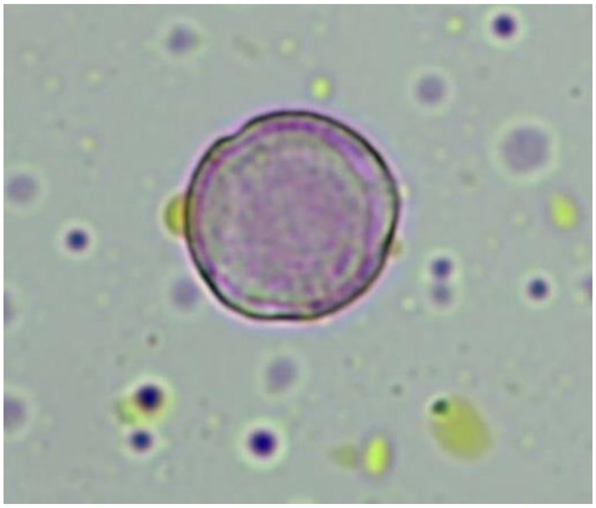

2. Label: *Malpighiaceae*

Description:

a. **Pollen unit:** Monad
b. **Size:** Large (14-15 μm)
c. **Polarity:** Isopolar
d. **Shape:** Spheroidal
e. **Aperture type:** Colporus
f. **Aperture condition:** Tricolporate
g. **Ornamentation:** Verrucate, scabrate

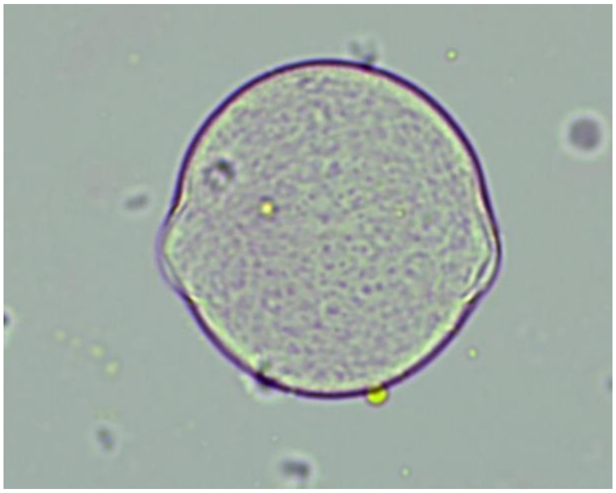

3. Label: *M1*

Description:

- **a. Pollen unit:** Monad
- **b. Size:** Large (18-27 μm)
- **c. Polarity:** Isopolar
- **d. Shape:** Spheroidal
- **e. Aperture type:**
- **f. Aperture condition:** Tricolporate
- g. **Ornamentation:** NA

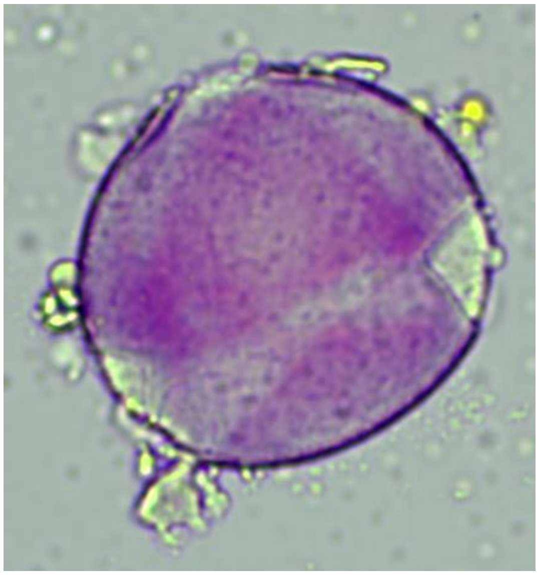

4. Label: *Combretaceae* sp.1

Description:

a. **Pollen unit:** Monad
b. **Size:** Small (8-13 μm)
c. **Polarity:** Isopolar
d. **Shape:** Spheroidal
e. **Aperture type:** Colporus
f. **Aperture condition:** Tricolporate
g. **Ornamentation:** NA

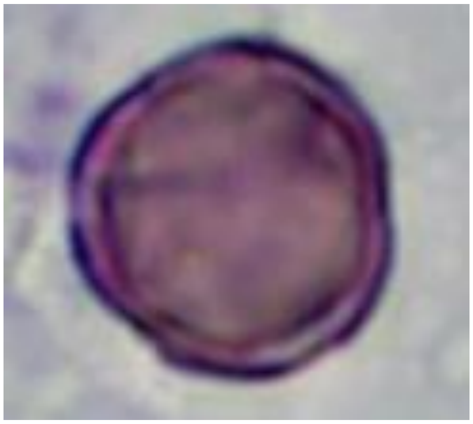

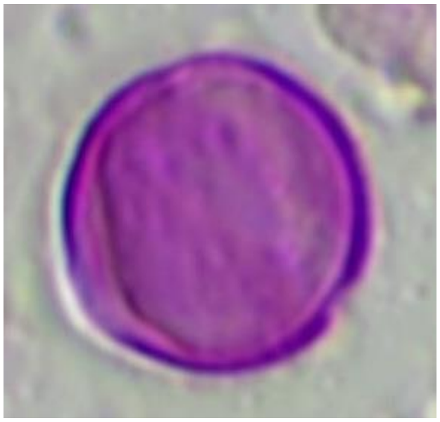

5. Label: *M3*

Description:

a. **Pollen unit:** Monad
b. **Size:** Small
c. **Polarity:** Isopolar
d. **Shape:** Spheroidal/Irregular infoldings
e. **Aperture type:** Colporus
f. **Aperture condition:**
g. **Ornamentation:** NA

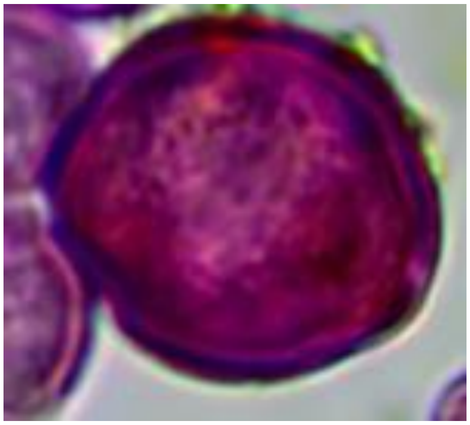

6. Label: *Fabaceae*

Description:

a. **Pollen unit:** Monad
b. **Size:** Small (μm)
c. **Polarity:** Isopolar
d. **Shape:** Spheroidal
e. **Aperture type:** Colporus
f. **Aperture condition:** Tricolporate
g. **Ornamentation:** Verrucate

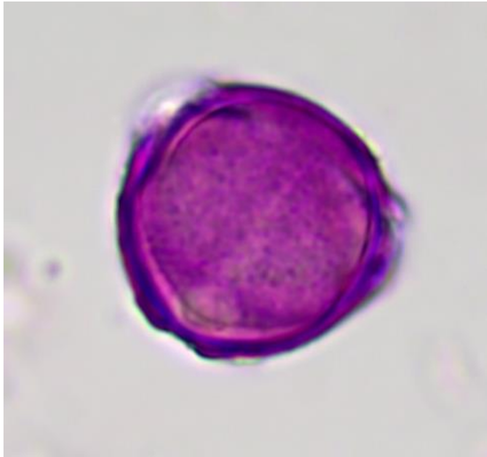

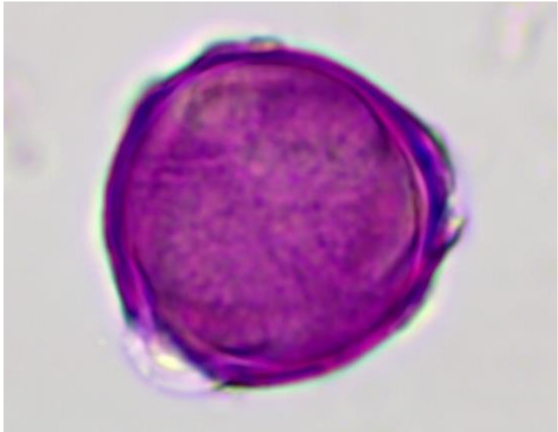

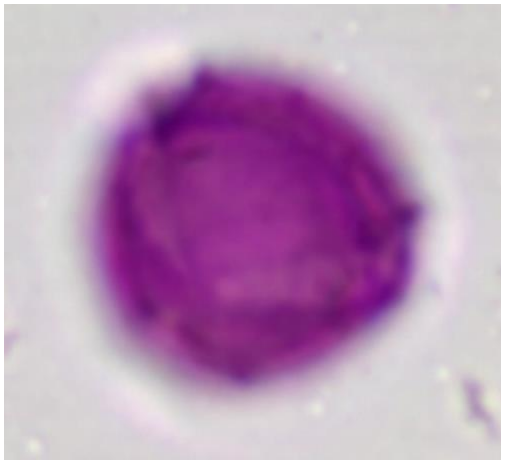

7. Label: *Asteraceae* sp.1

Description:

a. **Pollen unit:** Monad
b. **Size:** Small (μm)
c. **Polarity:** Isopolar
d. **Shape:** Spheroidal
e. **Aperture type:** Colporus
f. **Aperture condition:** Colporate
g. **Ornamentation:** Echinate

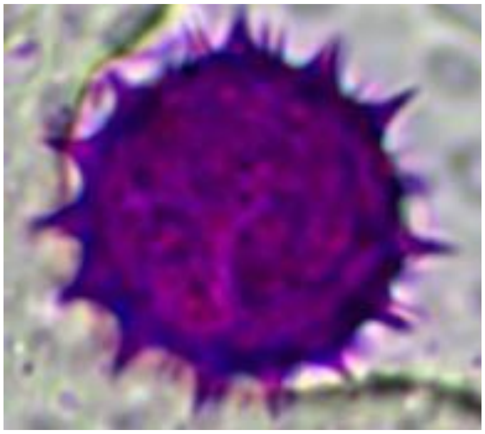

8. Label: *M5*

Description:

a. **Pollen unit:** Monad
b. **Size:** Small
c. **Polarity:**
d. **Shape:** Elliptic
e. **Aperture type:** Colporus
f. **Aperture condition:**
g. **Ornamentation:** Verrucate

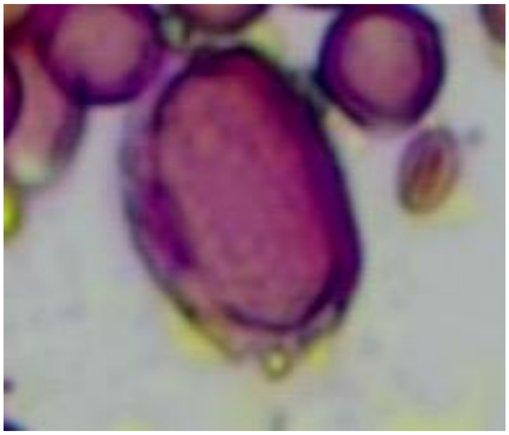

9. Label: *Passiflora* sp.1

Description:

a. **Pollen unit:** Monad
b. **Size:** Large (µm)
c. **Polarity:** Isopolar
d. **Shape:** Spheroidal
e. **Aperture type:** Ring-like aperture
f. **Aperture condition:** NA
g. **Ornamentation:** Reticulate

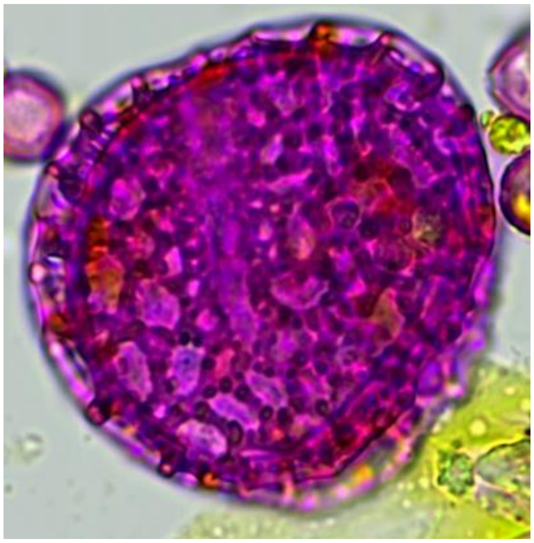

10. Label: *M6*

Description:

a. **Pollen unit:** Monad
b. **Size:** Large
c. **Polarity:** Isopolar
d. **Shape:** Spheroidal
e. **Aperture type:** Colporus
f. **Aperture condition:** Tricolporate
g. **Ornamentation:** NA

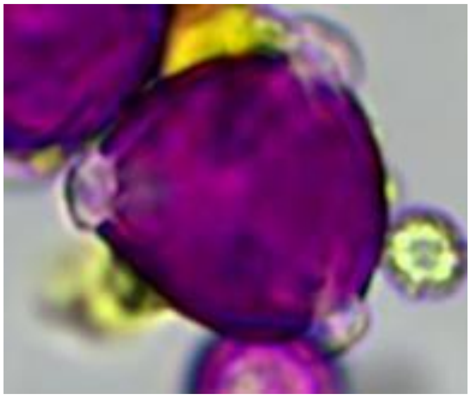

11. Label: *M7*

Description:

a. **Pollen unit:** Monad
b. **Size:** Small (9.5 – 20 μm)
c. **Polarity:** Isopolar
d. **Shape:** Triangular
e. **Aperture type:** Colporus
f. **Aperture condition:** Tricolporate
g. **Ornamentation:** NA

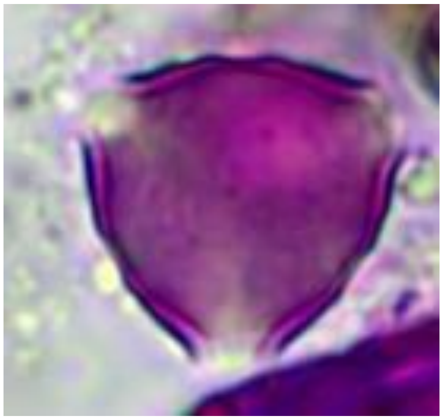

12. Label: *M8*

Description:

a. **Pollen unit:** Monad
b. **Size:** Large
c. **Polarity:** Isopolar
d. **Shape:** Spheroidal
e. **Aperture type:** Colporus
f. **Aperture condition:** NA
g. **Ornamentation:** Verrucate

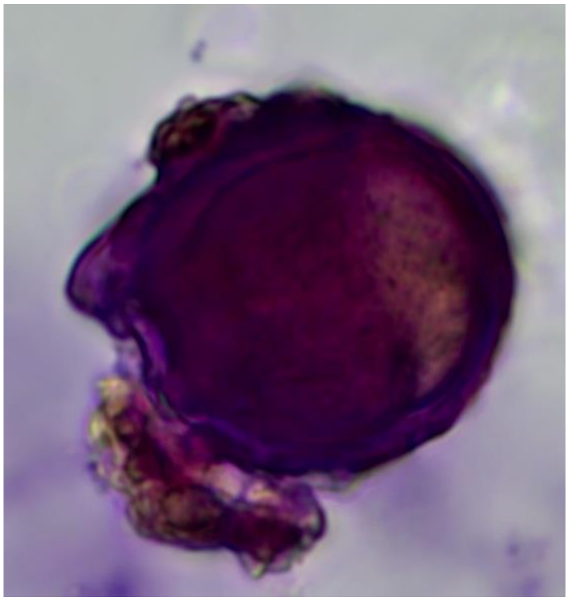

13. Label: *M9*

Description:

a. **Pollen unit:** Monad
b. **Size:** Large
c. **Polarity:** Isopolar
d. **Shape:** Spheroidal
e. **Aperture type:** Colporus
f. **Aperture condition:** NA
g. **Ornamentation:** Reticulate

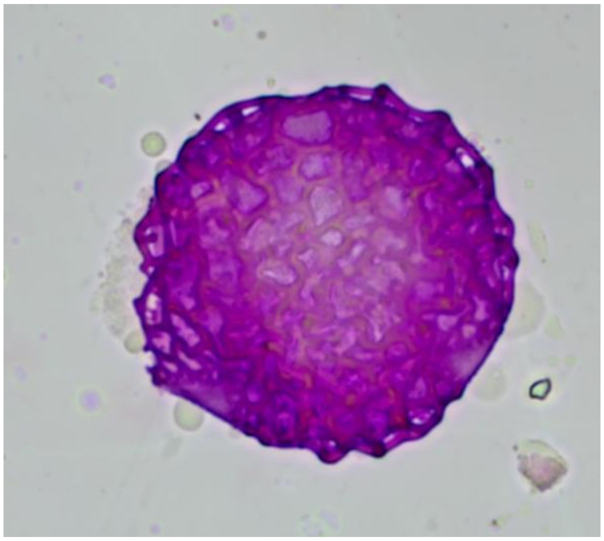

14. Label: *M10*

Description:

a. **Pollen unit:** Monad
b. **Size:** Small
c. **Polarity:** Isopolar
d. **Shape:** Spheroidal
e. **Aperture type:** Colporus
f. **Aperture condition:** Tricolporate
g. **Ornamentation:** NA

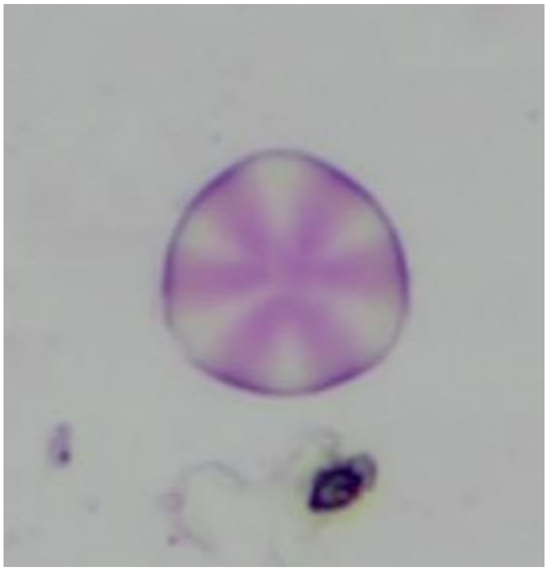

15. Label: *Asteraceae* sp.2

Description:

- **a. Pollen unit:** Monad
- **b. Size:** Small (13 – 18 μm)
- **c. Polarity:** Isopolar
- **d. Shape:** Spheroidal
- **e. Aperture type:** Colporus
- **f. Aperture condition:** Tricolporate, colporate
- **g. Ornamentation:** Echinate

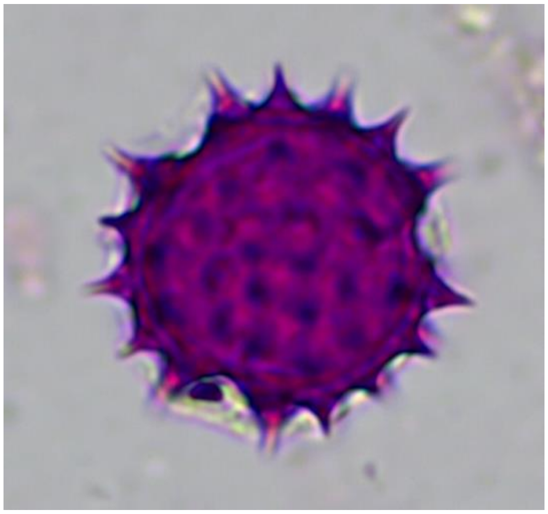

16. Label: *Solanaceae*

Description:

a. **Pollen unit:** Monad
b. **Size:** Small (12.5 – 14.5 μm)
c. **Polarity:** Isopolar
d. **Shape:** Elliptic
e. **Aperture type:** Colporus
f. **Aperture condition:**
g. **Ornamentation:** NA

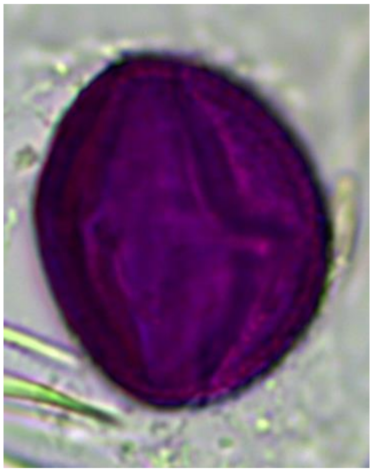

17. Label: *M12*

Description:

a. **Pollen unit:** Monad
b. **Size:** Large
c. **Polarity:** Isopolar
d. **Shape:** Spheroidal
e. **Aperture type:** Colporus
f. **Aperture condition:** Tricolporate
g. **Ornamentation:** NA

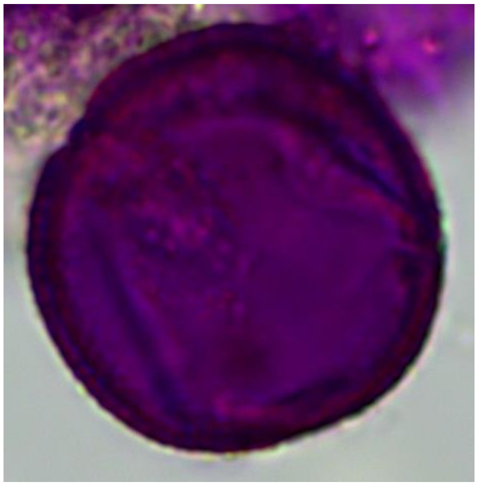

18. Label: *M13*

Description:

a. **Pollen unit:** Monad
b. **Size:** Large (12.3 – 15.2 μm)
c. **Polarity:** Isopolar
d. **Shape:** Spheroidal
e. **Aperture type:** Colporus
f. **Aperture condition:** Tricolporate
g. **Ornamentation:** Verrucate, scabrate

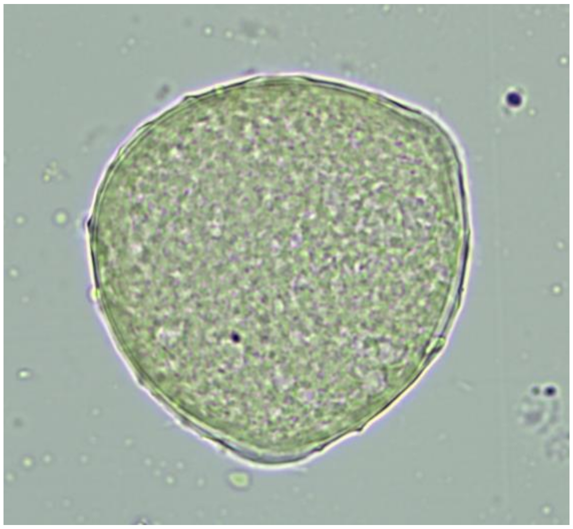

19. Label: *M14*

Description:

a. **Pollen unit:** Monad
b. **Size:** Small
c. **Polarity:** Isopolar
d. **Shape:** Spheroidal
e. **Aperture type:** Inaperture
f. **Aperture condition:** NA
g. **Ornamentation:** NA

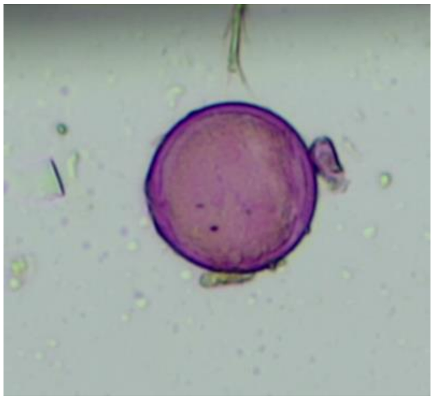

20. Label: *M15*

Description:

a. **Pollen unit:** Monad
b. **Size:** Small
c. **Polarity:** Isopolar
d. **Shape:** Spheroidal
e. **Aperture type:** Colporus
f. **Aperture condition:** NA
g. **Ornamentation:** NA

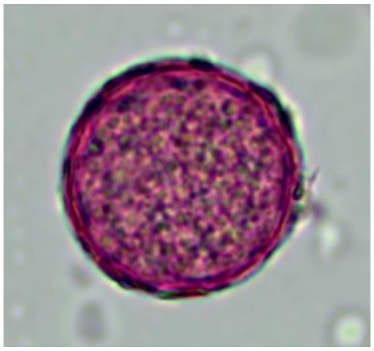

21. Label: *M16*

Description:

a. **Pollen unit:** Monad
b. **Size:** Large (22.3 – 40.8 μm)
c. **Polarity:** Isopolar
d. **Shape:** Spheroidal
e. **Aperture type:** Colporus
f. **Aperture condition:** NA
g. **Ornamentation:** Verrucate, gemmate, scabrate

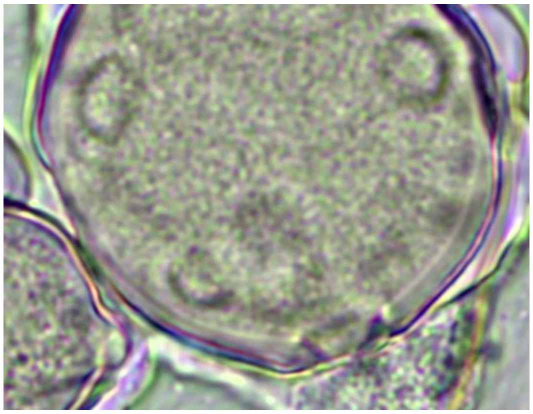

22. Label: *M17*

Description:

a. **Pollen unit:** Monad
b. **Size:** Small
c. **Polarity:** Isopolar
d. **Shape:** Elliptic
e. **Aperture type:** Colporus
f. **Aperture condition:**
g. **Ornamentation:** NA

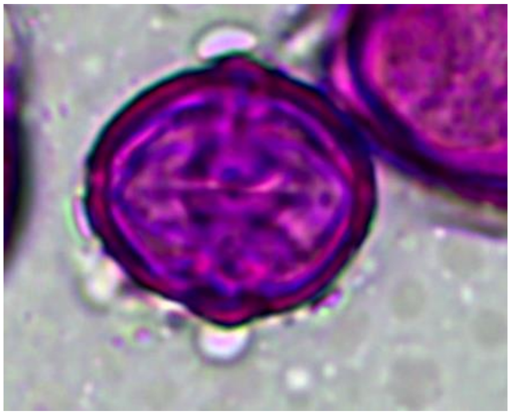

23. Label: *M18*

Description:

a. **Pollen unit:** Monad
b. **Size:** Small
c. **Polarity:** Isopolar
d. **Shape:** Elliptic/Infoldings
e. **Aperture type:** Colpus
f. **Aperture condition:**
g. **Ornamentation:** NA

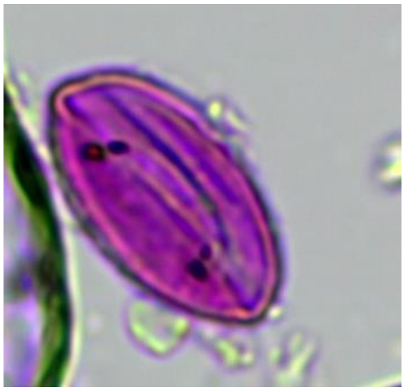

24. Label: *M19*

Description:

a. **Pollen unit:** Monad
b. **Size:** Medium
c. **Polarity:** Isopolar
d. **Shape:** Spheroidal
e. **Aperture type:** Colporus
f. **Aperture condition:** Pantoporate
g. **Ornamentation:** NA

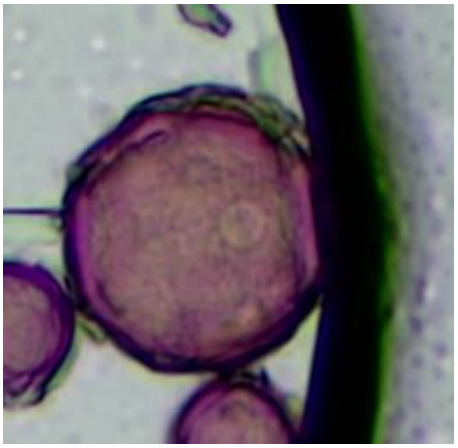

25. Label: *M20*

Description:

a. **Pollen unit:** Monad
b. **Size:** Small
c. **Polarity:** Isopolar
d. **Shape:** Elliptic
e. **Aperture type:** Colporus
f. **Aperture condition:** Tricolporate
g. **Ornamentation:** NA

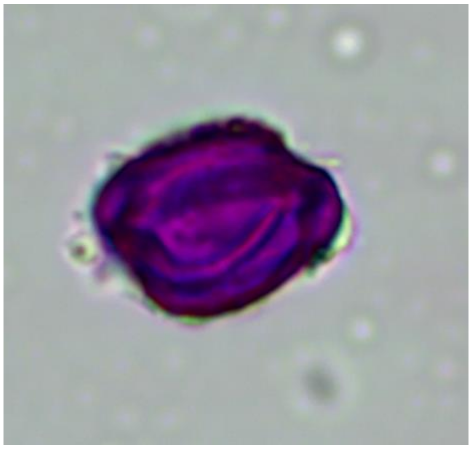

26. Label: *M21*

Description:

a. **Pollen unit:** Tetrad
b. **Size:** Small
c. **Polarity:** Heteropolar
d. **Shape:** Tetrad
e. **Aperture type:** Colporus
f. **Aperture condition:** NA
g. **Ornamentation:** NA

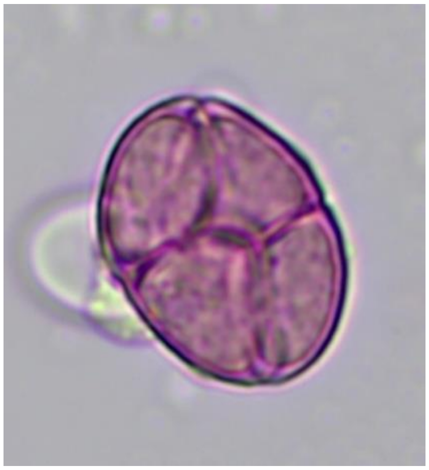

27. Label: *M22*

Description:

a. **Pollen unit:** Monad
b. **Size:** Small
c. **Polarity:** Isopolar
d. **Shape:** Spheroidal
e. **Aperture type:** Colporus
f. **Aperture condition:** Tricolporate
g. **Ornamentation:** Verrucate, scabrate

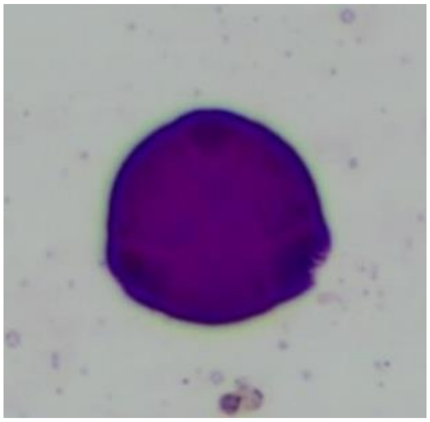

28. Label: *Combretaceae sp.2*

Description:

a. **Pollen unit:** Monad
b. **Size:** Small
c. **Polarity:** Isopolar
d. **Shape:** Spheroidal
e. **Aperture type:** Colporus
f. **Aperture condition:** Stephanocolporate
g. **Ornamentation:** NA

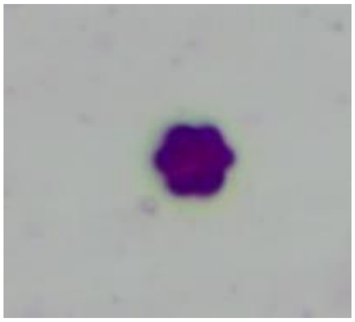

Supplement: Supplementary file 6 [file Presentation_2.pdf]
