# Supplementary material for: Gut microbiota variation of a tropical oil-collecting bee species far exceeds that of the honeybee
Source: Front Microbiol. 2023 May 17;14:1122489. doi: 10.3389/fmicb.2023.1122489 (PMC10229882; doi:10.3389/fmicb.2023.1122489)
Supplement: Supplementary file 7 [file Table_1.pdf]

Group 1	Group 2	H	p-value	q-value
**Comparison group 1:** ***Apis mellifera*** **vs.** ***Centris decolorata*** **(adults)**				
**Chao1**				
Apis mellifera **(n=6)**	***Centris decolorata*** **(n=10)**	0.011765	0.913627	0.913627
**Shannon**				
Apis mellifera **(n=6)**	***Centris decolorata*** **(n=10)**	7.952941	**0.004801**	0.004801
**Comparison group 2 :** ***Centris decolorata*** **females, males, larvae, and brood cell provisions**				
**Chao1**				
**gut** ***C. decolorata*** **female (n=6)**	**larva (n=2)**	1.000000	0.317311	0.425447
	**gut** ***C. decolorata*** **male (n=4)**	0.045455	0.831170	0.831170
	**brood cell provision (n=7)**	5.897959	**0.015158**	<u>0.090951</u>
**larva (n=2)**	**gut** ***C. decolorata*** **male (n=4)**	0.857143	0.354539	0.425447
	**brood cell provision (n=7)**	2.142857	0.143235	0.286470
**gut** ***C. decolorata*** **male (n=4)**	**brood cell provision (n=7)**	4.321429	**0.037635**	<u>0.112906</u>
**Shannon**				
**gut** ***C. decolorata*** **female (n=6)**	**larva (n=2)**	2.777778	0.095581	0.143371
	**gut** ***C. decolorata*** **male (n=4)**	1.136364	0.286422	0.343706
	**brood cell provision (n=7)**	9.000000	**0.002700**	0.016199
**larva (n=2)**	**gut** ***C. decolorata*** **male (n=4)**	0.214286	0.643429	0.643429
	**brood cell provision (n=7)**	4.200000	**0.040424**	<u>0.080848</u>
**gut** ***C. decolorata*** **male (n=4)**	**brood cell provision (n=7)**	7.000000	**0.008151**	0.024453

Supplementary Table 1. Results of alpha diversity differences analyses using Chao1 and Shannon indices. In bold are shown the significant results. The alpha diversity of comparison group 1 (*Apis mellifera* vs. *Centris decolorata*) shows significant differences in diversity using Shannon (p-value=0.004801) but no difference in richness using Chao1 (p-value=0.913627). Concerning comparison group 2 (*Centris decolorata* categories), brood cell provision microbiota shows significant differences both in diversity using Shannon (p-value=0.0027) and richness using Chao1 (p-value=0.008151) with other groups (gut of *C. decolorata* female and male). In addition, the brood cell provision microbiota also shows significant differences in terms of diversity using Shannon index with larva (p-value=0.040424). However, the q-values (adjusted p-values) that account for the percentage of false positive should rather be considered in the case of the larvae group, given its low sample size. All the q-value contradicting the p-value are underlined.

Attention of the reader is drawn to all the groups with samples size n<5 (sample size ≥5 being an assumption of Kruskal-Wallis test). For those samples, the H statistic is less well approximated to the chi-square distribution. Therefore, the null hypothesis (rejected when H is larger than the critical cutoff point in chi-square distribution) cannot be accurately tested. These results should thus be confirmed with greater sampling size.
