# Supplementary material for: Gut microbiota variation of a tropical oil-collecting bee species far exceeds that of the honeybee
Source: Front Microbiol. 2023 May 17;14:1122489. doi: 10.3389/fmicb.2023.1122489 (PMC10229882; doi:10.3389/fmicb.2023.1122489)
Supplement: Supplementary file 8 [file Table_2.pdf]

**Comparison group 1:** ***Apis mellifera*** **vs.** ***Centris decolorata*** **(adults)**		**Comparison group 2:** ***Centris decolorata*** **females, males, larvae, and brood cell provisions**	
**PERMANOVA results**		**PERMANOVA results**	
**method name**	PERMANOVA	method name	PERMANOVA
**test statistic name**	pseudo-F	test statistic name	pseudo-F
**sample size**	16	sample size	19
number of groups	2	number of groups	4
test statistic	8.971008	test statistic	3.832448
p-value	**0.001**	p-value	**0.001**
number of permutations	999	number of permutations	999
**PERMDISP results**		**PERMDISP results**	
**method name**	PERMDISP	method name	PERMDISP
**test statistic name**	F-value	test statistic name	F-value
**sample size**	16	sample size	19
number of groups	2	number of groups	4
test statistic	1.140257	test statistic	1.664951
p-value	0.112	p-value	0.190
number of permutations	999	number of permutations	999
**ANOSIM results**		**ANOSIM results**	
**method name**	ANOSIM	method name	ANOSIM
**test statistic name**	R	test statistic name	R
**sample size**	16	sample size	19
number of groups	2	number of groups	4
test statistic	0.964167	test statistic	0.720022
p-value	**0.001**	p-value	**0.001**
number of permutations	999	number of permutations	999

Supplementary Table 2. Results of beta diversity differences analyses using Permanova, Permdisp, and Anosim. In bold are shown the significant results. The beta diversity of comparison group 1 (*Apis mellifera* vs. *Centris decolorata*), as well as that of comparison group 2 (*Centris decolorata* categories) show significant differences using Permanova (both p-value=0.001), but Permdisp test shows that the observed differences in beta diversity could be explained by the difference in dispersion of Bray-Curtis distances (p-value=0.112 and p-value=0.190, respectively). Anosim however confirms significant differences in beta diversity between adults of both species, and between *Centris decolorata* categories (both p-value=0.001).
